# G-quadruplex formation in RNA aptamers selected for binding to HIV-1 capsid

**DOI:** 10.3389/fchem.2024.1425515

**Published:** 2024-10-22

**Authors:** Miles D. Mayer, Margaret J. Lange

**Affiliations:** ^1^ Department of Molecular Microbiology and Immunology, Columbia, MO, United States; ^2^ Department of Biochemistry, Columbia, MO, United States

**Keywords:** thioflavin T, circular dichroism, thermodynamics, molecular tool, non-canonical

## Abstract

HIV-1 capsid protein (CA) is essential for viral replication and interacts with numerous host factors to facilitate successful infection. Thus, CA is an integral target for the study of virus-host dynamics and therapeutic development. The multifaceted functions of CA stem from the ability of CA to assemble into distinct structural components that come together to form the mature capsid core. Each structural component, including monomers, pentamers, and hexamers, presents a variety of solvent-accessible surfaces. However, the structure-function relationships of these components that facilitate replication and virus-host interactions have yet to be fully elucidated. A major challenge is the genetic fragility of CA, which precludes the use of many common methods. To overcome these constraints, we identified CA-targeting aptamers with binding specificity for either the mature CA hexamer lattice alone or both the CA hexamer lattice and soluble CA hexamer. To enable utilization of these aptamers as molecular tools for the study of CA structure-function relationships in cells, understanding the higher-order structures of these aptamers is required. While our initial work on a subset of aptamers included predictive and qualitative biochemical characterizations that provided insight into aptamer secondary structures, these approaches were insufficient for determining more complex non-canonical architectures. Here, we further clarify aptamer structural motifs using focused, quantitative biophysical approaches, primarily through the use of multi-effective spectroscopic methods and thermodynamic analyses. Aptamer L15.20.1 displayed particularly strong, unambiguous indications of stable RNA G-quadruplex (rG4) formation under physiological conditions in a region of the aptamer also previously shown to be necessary for CA-aptamer interactions. Non-canonical structures, such as the rG4, have distinct chemical signatures and interfaces that may support downstream applications without the need for complex modifications or labels that may negatively affect aptamer folding. Thus, aptamer representative L15.20.1, containing a putative rG4 in a region likely required for aptamer binding to CA with probable function under cellular conditions, may be a particularly useful tool for the study of HIV-1 CA.

## 1 Introduction

The HIV capsid protein (CA) is involved in or facilitates several steps of viral replication, including assembly (Lei et al., 2023; Banerjee and Voth, 2024), maturation (Ganser-Pornillos et al., 2008; Rossi et al., 2021), trafficking through the cytosol to the nucleus (Toccafondi et al., 2021; Deshpande et al., 2023; Ingram et al., 2024; Ananth et al., 2024; Hudait and Voth, 2024), reverse transcription (Zila et al., 2021; Burdick et al., 2024; Ananth et al., 2024), and integration (Zila et al., 2021; Ingram et al., 2024). During virus assembly, CA is a component of the Gag and GagPol polyproteins, which are processed by the viral protease during maturation, liberating CA to assemble into distinct structural components that form the mature capsid core, ultimately composed of approximately 250 hexamers and 12 pentamers, which assemble through various intermediates (Ganser-Pornillos et al., 2008; Rossi et al., 2021).

It is well understood that CA structure is critically important to the multifaceted functions of CA. Indeed, CA is highly conserved across HIV clades and mutations that alter the relative stability of CA produce severe infectivity defects due to disruption of one or more replication events (Rihn et al., 2013). Thus, the capsid is genetically fragile, while also maintaining a high degree of structural plasticity required for its functional repertoire. However, this genetic fragility presents several challenges to understanding the role and interactions of CA during replication, as common technical approaches including mutation and protein tagging disrupt many replication steps (Francis et al., 2022; Rihn et al., 2013). To address these challenges, we have previously identified aptamers that bind different CA structural forms.

Aptamers are single-stranded nucleic acids selected to bind to a target of interest with high specificity and affinity. The exceptional dynamic folding characteristics of nucleic acids make them spectacular tools, having been useful for the detection of small molecules (Win et al., 2006), nucleic acids (Dai et al., 2024), proteins (Whitehouse et al., 2024), and other biomolecules (Liu et al., 2024) *in vitro* and *in vivo* (Ong et al., 2017). Of particular relevance for CA, aptamers are capable of discriminating different conformations of the same protein (Yunn et al., 2021; Sarell et al., 2014). Our goal is to develop CA-binding aptamers as tools to better understand CA structure and function in cells. Importantly, aptamers are easily modified for a variety of applications (Proske et al., 2005; Gao et al., 2016), including sensing and imaging techniques and affinity purification. However, identifying sites amenable to modification is important to avoid altering native folding, which may negatively affect binding (Zhao et al., 2014). Thus, determining aptamer structure is of particular importance in modifying aptamers for downstream applications.

We previously initiated preliminary studies aimed at resolving the structure for a panel of four aptamers identified in our selections against CA (Gruenke et al., 2022; Gruenke et al., 2024). These studies utilized enzymatic probing, generation of covariation models, mutational profiling, nuclear magnetic resonance (NMR), and limited thermodynamic analysis. Our investigation highlighted several challenges in the determination of aptamer RNA structure, presenting an opportunity to optimize structure determination with respect to cost and time and to perform more in-depth studies to better understand the thermodynamic properties and molecularity of our aptamers, including an aptamer for which our initial data strongly suggested the presence of a G-quadruplex structure (Gruenke et al., 2024).

G-quadruplexes (G4s) are non-canonical secondary structures formed through two or more stacks of G- or mixed-tetrads. They have been studied extensively and are thought to exist in human and viral genomes (Hänsel-Hertsch et al., 2017; Lavezzo et al., 2018), potentially playing roles in genes and their regulation (Phan et al., 2007; Lin et al., 2012; Bay et al., 2017; Ceschi et al., 2020). G4s may be distinctly polymorphic (Kypr et al., 2009; Malgowska et al., 2014; Largy et al., 2016), with different conformations dependent upon any number of variables (Largy et al., 2016; Pavc et al., 2020; Zhao et al., 2014; Nishio et al., 2021) existing and co-existing based on internal and external factors (Lech et al., 2012; Dai et al., 2007; Malgowska et al., 2014; Webba da Silva et al., 2009; Hazel et al., 2004; Zhang et al., 2009; Laughlan et al., 1994; Balthasart et al., 2013; Kolesnikova and Curtis, 2019; Tang and Shafer, 2006; Víglaský et al., 2010). RNA G4s (rG4), which are distinct from DNA G4s due to the ribose 2′hydroxyl groups affecting possible configurations, have been shown to vary in cooperativity, reliance on potassium, and number of transition states depending upon the number of G’s in each tract (Mullen et al., 2012), where repeats of less than 3 G’s each tend to result in rG4s with fewer transition states, higher requirements for potassium, more positive cooperativity, bimolecularity (Collie et al., 2010; Martadinata and Phan, 2013; Tang and Shafer, 2006), and overall complexity (Xiao et al., 2017).

The same methods used for determining other RNA architectures can be utilized to determine rG4 structures. However, more direct methods (Laughlan et al., 1994; Chung et al., 2015) often require more time and significant sample amounts, resulting in issues like crowding that could force specific conformations (Malgowska et al., 2016), and may not be useful in identifying highly polymorphic G4s (Passalacqua et al., 2023). Fortunately, due to their unique nature, some cost- and time-effective methods exist that can assist in determining the likelihood of a G4 in an oligonucleotide and potentially provide additional structural information (Chung et al., 2014). Circular Dichroism (CD) and UV-Visible (UV-Vis) spectrophotometry are often employed when studying G4s, and the data collected from these methods may be manipulated to perform folding and thermodynamic calculations (Williams et al., 2001; Mergny et al., 2005; Mergny and Lacroix, 2009; Gray and Chaires, 2012; Steger, 1994; Greenfield, 2006). Further, more recent methods utilizing G4-specific small molecules have been employed in biochemical assays for detection and study of G4s (Mendes et al., 2022; Xu et al., 2016; Renaud de la Faverie et al., 2014). Together, these methods are efficacious in identifying the potential existence of rG4s, as well as limited topological information (Chung et al., 2015; Chung et al., 2014), general degree of polymorphism (Malgowska et al., 2014; Kypr et al., 2009), and estimates of molecularity (Gralla and Crothers, 1973; Marky and Breslauer, 1987).

Aptamers containing G4s have been modified and employed successfully (Bock et al., 1992; Paige et al., 2011; Zhao et al., 2014; Kovačič et al., 2020; Zheng et al., 2021), and may be uniquely useful as tools for studying targets of interest through techniques that are G4-specific, potentially reducing requirements for modifications or labels that could interfere with native conformation (Saccà et al., 2005). Thus, G4-based aptamers may not require additional experimentation to determine necessary binding regions and, as functional label-free aptamers, could be useful under both *in vitro* and *in vivo* conditions. However, an overall understanding of aptamer structure is necessary, not only to identify possible regions for modification, but to verify the existence of G4 motifs under specific conditions and their potential as signaling moieties.

Here we continue our structural studies of four representative aptamers previously selected to bind CA, termed L15.6.1, L15.7.1, H7.10.1, and L15.20.1, with a focus on aptamer L15.20.1 (Gruenke et al., 2022; Gruenke et al., 2024). We apply multiple approaches to justify the existence of a putative rG4 architecture within L15.20.1 and speculate on topological details utilizing cost- and time-effective approaches in the hope that upcoming work identifying necessary RNA elements for CA binding may be improved.

## 2 Materials and methods

### 2.1 DNA, RNA, PCR, *In vitro* transcription and Urea-PAGE

DNA and RNA (ADAM10 and its variants) sequences were purchased from Integrated DNA Technologies (IDT; Coralville, IA.) and Genscript Biotech (Piscataway, NJ), and can be found in Supplementary Table S1. For DNA sequences, PCR was performed and both aptamer sequence and purity were initially confirmed through Sanger Sequencing (MU Genomics and Technology Core; Columbia, MO.). DNA was converted to RNA using a mutated T7 Polymerase (Y639F) for *in vitro* transcription at 37°C. 6% PAGE with 8 M urea gels were used for separation and denaturation of each aptamer. 300 mM sodium acetate was used for aptamer gel purification under rotating conditions in a cold room (4°C) for 3 days, followed by precipitation using anhydrous ethanol, where pelleting through centrifugation was performed at 16,100 × g at 4°C for 30 min. RNA was stored at −20°C in nuclease-free water prior to use. L15.20.1 mutants (L15.20.1_1A and L15.20.1_2A) were produced using multiple DNA sequences with incorporated mutations that were first annealed and then subjected to extension and amplification using Taq polymerase. RNA from these sequences was otherwise prepared in the same manner as above.

### 2.2 CD spectrophotometry, UV-Melting curves, and thermal difference spectra

CD experiments were performed using a Jasco J-1500 spectrophotometer with an MPTC-511 Peltier 6-Position cell holder and was utilized as previously discussed (Gruenke et al., 2024). Briefly, nucleic acids were denatured at 95°C for 1 min and placed into folding buffer comprised of 50 mM Tris, 150 mM XCl (where X = K^+^ or Li^+^), and 1 mM MgCl_2_ at a concentration of 10 µM and slowly cooled to room temperature to allow for refolding. CD experiments were performed using four accumulations per measurement. Spectra were collected from 340 nm to 200 nm at a speed of 200 nm/min. Values were initially confirmed to be at an absorbance near 0.8; a starting value that provides good signal while also remaining in the linear range of absorbance when shifting to higher temperatures, as hyperchromicity occurs at most wavelengths used to measure nucleic acids as they unfold. To account for any chromicity shifts, absorbance values were monitored throughout measurements to ensure a linear range (with a maximum absorbance of ∼1.2 within ranges of interest). Similarly, the high-tension voltage was kept at 500 V or less within the spectral ranges of interest. CD spectra were adjusted based on standard curves for aptamers under their respective cationic conditions. Spectra for refolding and Mg^2+^ reliance were previously collected (Gruenke et al., 2024).

Thermal difference spectra (TDS) for each aptamer, as well as the rG4 positive control, ADAM10, were produced by subtracting the absorbance values of the folded form(s) from the absorbance values of the unfolded form(s) for each representative. Utilized values were collected from 340 nm to 200 nm at temperatures determined by chromicity data, respective to their presumed folded and unfolded structures.

Melting curves for the aptamers and the rG4 control, ADAM10, were collected using the J-1500 spectrophotometer, where measurements were collected from 0°C to 95°C with a 3°C/min ramp. This method is otherwise the same as that performed for the CD measurements previously discussed. These curves were used to analyze chromicity shifts at 260 nm and 295 nm for each representative. Further, derivative plots were formed at 260 nm for each aptamer as a way to confirm T_m_ values and assist in roughly identifying molecularity of L15.20.1 through comparison of thermodynamic data and derivative spectra (Gralla and Crothers, 1973; Marky and Breslauer, 1987; Schroeder and Turner, 2009) along with folding plots.

Hysteresis kinetic data was collected using both fast and slow methods to examine potential variations in refolding. Data was collected similarly to other CD experiments, however, the faster method employed a 3°C/min gradient with 5°C measurement intervals and a 15 s wait time, while the slower method collected data at a gradient of 1°C/min, 2°C measurement intervals, and a 90 s wait time. Data was plotted to examine folding at 265 nm.

### 2.3 Folding and thermodynamic calculations

Ellipticity ( θ ) data collected during thermal denaturation studies of aptamer and control representatives provided *α* values under the utilized cationic conditions, where $\theta_{t}$ is the ellipticity of the aptamer being studied at a selected temperature, $\theta_{U}$ is the ellipticity of the unfolded aptamer, and $\theta_{F}$ is the ellipticity of the folded aptamer. The data collected at 265 nm was plotted from 0°C to 95°C and fitted through non-linear regression, allowing for determination of melting temperatures (T_m_) for each aptamer, where α = 0.5. These values also enabled assessment of the general fold at a given temperature, where (Greenfield, 2006):

$$\alpha =\frac{\left(\theta_{t}-\theta_{U}\right)}{\left(\theta_{F}-\theta_{U}\right)}$$

As previously discussed, derivative plots were also produced to compare with the calculated T_m_ values. The folding equilibrium constants ( K ) calculated for the determination of free folding energies ( ΔG ) for each aptamer under different cationic conditions were collected utilizing the following (Greenfield, 2006; Hannoush and Damha, 2001):

$$K=\frac{\alpha }{\left(1-\alpha \right)}$$

ΔG=−RTlnK

Derived van ’t Hoff equations and plots were utilized to expand our understanding of the thermodynamic properties of each aptamer, including free energy, enthalpy, and entropy values for both systems and individual temperatures under variable cationic conditions (Hannoush and Damha, 2001; Greenfield, 2006):

$$\ln\left(\frac{K_{2}}{K_{1}}\right)=\frac{\Delta H}{R}\left(\frac{1}{T_{1}}-\frac{1}{T_{2}}\right)$$

ΔG=ΔH – TΔS

Molar extinction coefficients were also utilized and were determined through absorbance measurements at 260 nm collected with both a NanoDrop One and Jasco J1500 spectrophotometer and calculated with the following (Del Villar-Guerra et al., 2017):

$$\varepsilon_{F}=\varepsilon_{U}\frac{A_{F}}{A_{U}}$$

### 2.4 Statistical analysis

Statistical, fitting, and regression analyses were performed using GraphPad Prism for all relevant data presented here and within the Supplementary Material.

### 2.5 Thioflavin T fluorescence assays

Thioflavin T (ThT) Assays were performed with Greiner Bio One black 96 well plates (Lot E1212ØØN). ThT (2390-54-7) was purchased from Sigma Aldrich. A CLARIOstar Plus High-Performance Plate Reader (BMG Labtech) was utilized for fluorescence detection. ThT-Nucleic acid complexes were excited at a wavelength of 440 nm (bandpass of 15 nm) and emission values were collected at a wavelength of 487 nm (bandpass of 20 nm) with a dichroic filter set at 462.2 nm.

Unfolded nucleic acids were heated to 90°C in 50 mM Tris, 150 mM KCl, and 1 mM MgCl_2_ to replicate physiological conditions and slowly cooled to room temperature to induce folding. Nucleic acid and ThT concentrations were 2 µM and 1 μM, respectively, and each well contained a total volume of 20 µL.

Nucleic acids were normalized based on their respective calculated molar extinction coefficients (Del Villar-Guerra et al., 2017) using both a NanoDrop One and Jasco J-1500 spectrophotometer with an MPTC-511 Peltier 6-Position cell holder under the above buffer conditions, save for cationic variations.

Further, to more conclusively determine differences between noise and structural components, as well as to potentially identify the necessity of certain aptamer regions for the formation of rG4 moieties, complementary oligos and aptamer mutants were utilized as additional controls beyond the standard positive and negative nucleic acid controls. Complementary oligo concentrations were also calculated using molar extinction coefficients, and equimolar amounts of aptamer or control and complementary oligo were combined and treated in the same fashion as above.

## 3 Results

### 3.1 CD spectrophotometry

CD spectrophotometry is a useful instrumental method for identifying certain molecular architectures and has been widely used to assist in characterizing protein and nucleic acid secondary structures. However, when considering the aptamers being studied, which are single-stranded RNA molecules comprised of 104 bases, it is important to note that as the size and complexity of the overall structure and the potential for multiple folded forms increases, the amount of information obtained may become more limited, as all forms will contribute to the overall spectrum collected. Nonetheless, the approach is applicable if interested in determining particular moieties within the biomolecule being assessed.

One structure that is often identified, in part, using CD, is the rG4. Nucleic acids are generally examined from around 200 nm–320 nm, with parallel rG4 moieties presenting positive peaks at 265 nm and negative peaks at 240 nm. Though rG4 structures share similar spectra to A-form duplexes, rG4 secondary structures can be ascertained, to some extent, by increasing the ionic concentration of potassium, which stabilize rG4s. Increases in ellipticity at 265 nm can be visualized in the CD spectra, which is relatively unique to these motifs.

**FIGURE 1:**
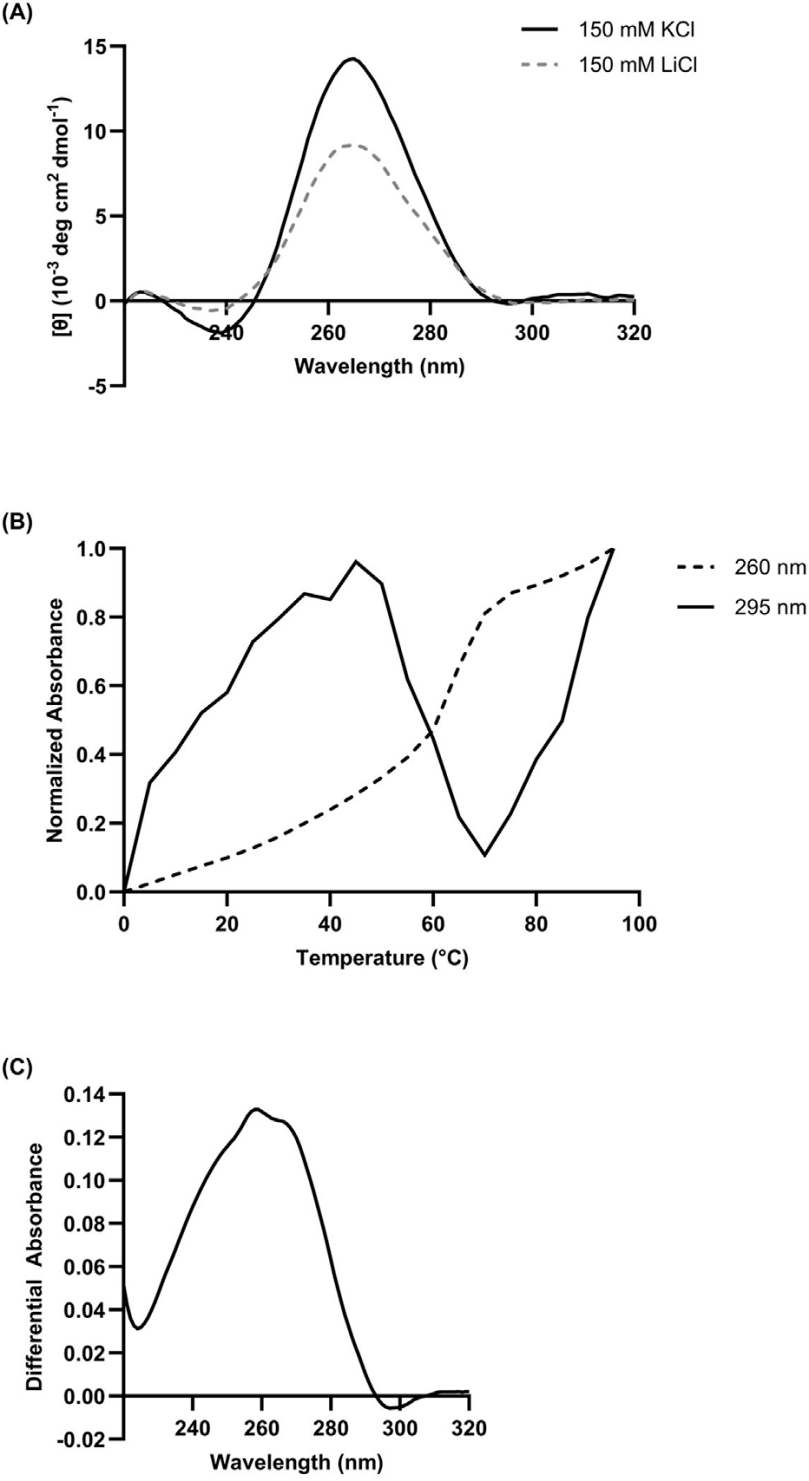
**(A)** Composite CD spectrum (n = 3) of 10 µM L15.20.1 under high lithium and high potassium conditions, with an expected negative at ∼240 nm and an expected positive at ∼265 nm. Solution buffer: 50 mM Tris, 150 mM XCl (X = K^+^ or Li^+^), 1 mM MgCl_2_. **(B)** Chromicity plot at 260 nm and 295 nm and **(C)** TDS of 10 µM L15.20.1 under high potassium conditions (50 mM Tris, 150 mM KCl, 1 mM MgCl_2_); n = 3.

As our previous work provided CD and NMR data indicating potential rG4 formation (Gruenke et al., 2024), particularly in representative L15.20.1, replicates of CD spectrophotometric data were collected for this aptamer, both to confirm previous results and provide some of the necessary data to calculate quantitative thermodynamic and folding values. In the absence of K^+^ and presence of Li^+^, L15.20.1 provided spectra indicative of a folded RNA structure. However, under high K^+^ conditions, ellipticity at 265 nm increased significantly (Figure 1A), confirming previous qualitative CD measurements. The CD spectra for two of the other three representatives (L15.7.1 and H7.10.1) also previously showed increases in ellipticity at this wavelength. In L15.6.1, which is expected to have primarily loop motifs, no signal change at 265 nm was seen when varying ionic concentrations. From these data we can reasonably conclude that an rG4 motif exists in L15.20.1 and appears to be highly reliant on ionic conditions. However, to clarify the veracity of these results and fidelity of the method, we also incorporated a positive control known to be a highly stable three-tetrad rG4, ADAM10, which was examined (Supplementary Figure S1A) and spectrally fit with previous work (Lammich et al., 2011), indicating our instrumentation and measurement approaches were acceptable and reproducible.

### 3.2 UV-Vis Spectrophotometry

#### 3.2.1 Chromicity studies

Nucleic acids are generally measured at a wavelength of 260 nm and show an increase in absorbance as temperature increases, referred to as hyperchromicity. As they are denatured, the higher-order structures melt and hydrogen bonding between bases cease to exist leaving functional groups freely available to absorb light, exciting the electrons of bases to higher energy states, resulting in the overall signal received at the detector decreasing. However, in the case of certain non-canonical moieties, particularly the G4, absorbance at slightly higher wavelengths, most often 295 nm, appears to decrease. This is considered a hypochromic shift in absorbance and is thought to occur due to inter- and intra-molecular interactions, particularly non-canonical types of π-stacking and hydrogen bonding, light-induced dipole forces, and both the n-π* and π-π* transitions of individual and interacting bases (Tinoco, 1960; Danilov, 1974; Mergny et al., 2005).

Spectrophotometric analyses of L15.20.1 at 260 nm under high potassium conditions strongly indicated a hyperchromic shift; an expected result for nucleic acids at this wavelength. At 295 nm, the plot showed a significant hypochromic shift between 45°C and 70°C indicative of a non-canonical structure, fitting with the rG4 prediction (Figure 1B). However, as this does not occur throughout the entirety of the plot, it may indicate the potential for at least two major contributors to the spectra dependent on temperature. Under high lithium conditions, L15.20.1 appears fairly hyperchromic at both wavelengths (Supplementary Figure S2A). The spectra for the other three representatives under high potassium conditions at 260 nm were also hyperchromic in nature, while at 295 nm, lulls can be seen but are still consistently hyperchromic throughout the majority of the spectrum (Supplementary Figure S3). To demonstrate the applicability of hypochromicity measurements as a method of rG4 detection, ADAM10 was also examined. Previous work has shown that, above 50 μM KCl, ADAM10 will not fully melt (Lammich et al., 2011), indicating that the highly stable nature of the rG4 under high potassium conditions may result in significantly increased melting temperatures. We confirmed this to be the case, but nonetheless, hypochromicity can be visualized for the nucleic acid (Supplementary Figure S1B).

#### 3.2.2 UV thermal denaturation

TDS are often utilized in conjunction with CD, NMR, chromicity spectra, as well as folding and thermodynamic calculations to determine the existence of non-canonical structures such as the rG4 (Kabbara et al., 2022), though they can be used to obtain information for other nucleic acid structures as well (Mergny et al., 2005). In the case of G-tetrads, positive peaks are often seen at ∼240 nm and ∼270 nm (Mergny et al., 2005). However, data in these other regions, from 240 nm to 270 nm, will likely not be useful when considering larger oligonucleotides with multiple secondary motifs and many potential conformations that may play a role in resultant signal or spectra. Nonetheless, obtaining absorbance data as a nucleic acid of interest is denatured may provide indicators of certain secondary structures, conformational changes, and related transitions from the folded to unfolded state (Mergny et al., 2005). These spectra are formed through the simple subtraction of folded absorbance values from denaturing values. Though G-tetrads have potential indicators in the positive regions of a TDS, these may not be seen when examining G4s that exist among other motifs. However, G4s are expected to display hypochromicity at 295 nm, indicating that a TDS spectrum of a nucleic acid containing one is anticipated to have a negative region in that wavelength range.

TDS were prepared from UV-Vis spectrophotometric data for each representative. The TDS for aptamer L15.20.1 under high potassium conditions shows a distinct hypochromic shift at 295 nm, indicating the presence of a non-canonical structure such as a G-tetrad, and consistent with expected spectra for an rG4 (Figure 1C), as compared to high lithium, where no negative is seen near 295 nm (Supplementary Figure S2B). TDS for the other three representatives were also collected (Supplementary Figure S4). Neither the L15.7.1 nor the H7.10.1 spectra provide strong indications for rG4s, with no clear hypochromic shifts. This is one of a few analyses that led to the selection of L15.20.1 as the focus of this work. L15.6.1, which is not predicted to contain an rG4, did not provide any indications that would contradict this expectation. To clarify that a negative value at 295 nm is indicative of an rG4, ADAM10 was also examined, showing positive peaks at ∼240 nm and ∼270 nm, as is typical for an rG4 with no other secondary motifs, and displays a trough at 295 nm as expected (Supplementary Figure S1C).

#### 3.2.3 Derivatives and melting

First and second derivatives can be calculated at a wavelength of interest (generally at 260 nm for nucleic acids) and are often used to estimate the T_m_ values of nucleic acids. While this is one of many ways to estimate T_m_, generally a combination of methods, such as through the use of the nearest neighbors method, is preferable. Further, past thermodynamic studies have theorized that the peak of a derivative plot will fit the T_m_ calculated through folding and van ‘t Hoff analyses only if the nucleic acid is unimolecular in nature (Gralla and Crothers, 1973; Marky and Breslauer, 1987; Schroeder and Turner, 2009). It should be noted that these previous experiments were performed on much smaller nucleic acids, and the increasing complexity that comes with these much larger aptamers makes the application of these conclusions less reliable. Nonetheless, experiments with these aptamers reveal that derivative plots and thermodynamic calculations can vary significantly and may provide some insight into the molecularity of the aptamers under different cationic conditions.

**FIGURE 2:**
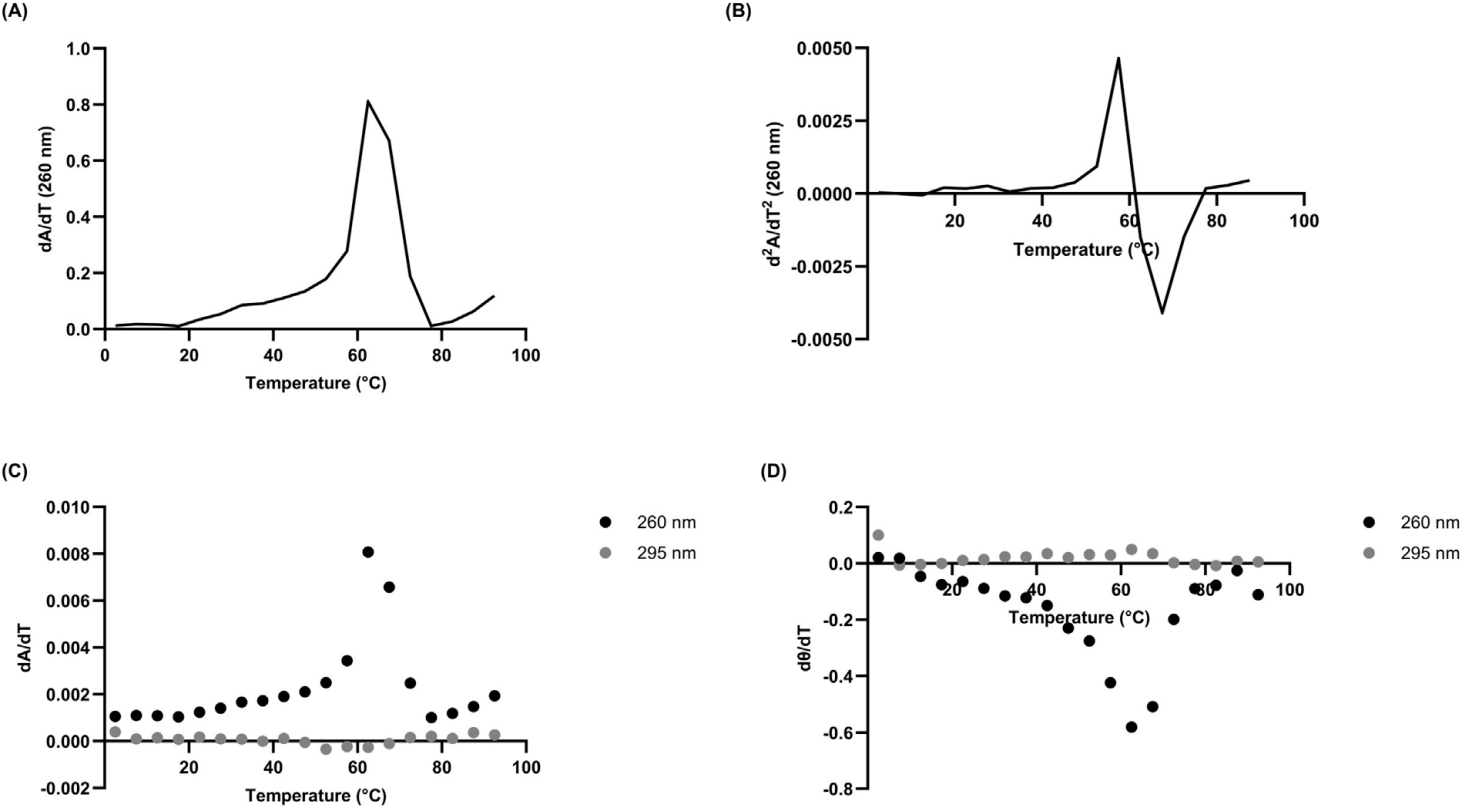
Derivatives of 10 µM L15.20.1 under high potassium conditions (50 mM Tris, 150 mM KCl, 1 mM MgCl_2_); n = 3. **(A)** Absorbance first derivative plot with peak near 61°C. **(B)** Absorbance second derivative plot at 260 nm clarifying the T_m_ just above 61°C at y = 0. **(C)** Absorbance first derivative plot at 260 nm and 295 nm. **(D)** Ellipticity first derivative plot at 260 nm and 295 nm.

In the case of L15.20.1 under high potassium conditions at 260 nm, the derivative plots and calculated T_m_ values are 61°C and 59.89 ± 1.829°C, respectively (Figure 2; Supplementary Table S2). This may imply an overall unimolecular nature to the aptamer when potassium is available. Under high lithium conditions, however, L15.20.1 shows a larger variation of around 10°C between derivative values and calculated T_m_ (Supplementary Tables S2, S3; Supplementary Figure S5). For L15.6.1 the calculated T_m_ and derivative values under high lithium and potassium conditions are within 1°C (Supplementary Figures S6A, S7A; Supplementary Table S3). For L15.7.1, however, under high lithium conditions, results vary by almost 5°C, while under high potassium conditions, the values are within 1.5°C of each other (Supplementary Figures S6B, S7B; Supplementary Table S3). In the case of H7.10.1, under high lithium conditions, the differences between calculated T_m_ and derivative values are drastic, with a variation of more than 15°C. However, under high potassium conditions, the variation is around 1.5°C (Supplementary Figures S6C, S7C; Supplementary Table S3). Considering that L15.6.1 has shown little difference in any measured parameter, it is possible that the aptamer is unimolecular in nature. Aptamer L15.7.1, which does provide some indications of a non-canonical G-rich structure, appears to vary little when under high potassium conditions. The same can be said for H7.10.1. However, it should be clearly noted that, with larger oligonucleotides, many methods generally applied assuming a two-state model may not be as accurate due to the increased number of interactions in any given nucleic acid. Our rG4 positive control, ADAM10, provides a good example of this, as under high potassium conditions, derivative plots indicate multiple values (not shown), and thus do not fit a two-state model under these circumstances. However, under high lithium conditions, a single derivative peak exists (not shown), and a two-state model could be applied.

**FIGURE 3:**
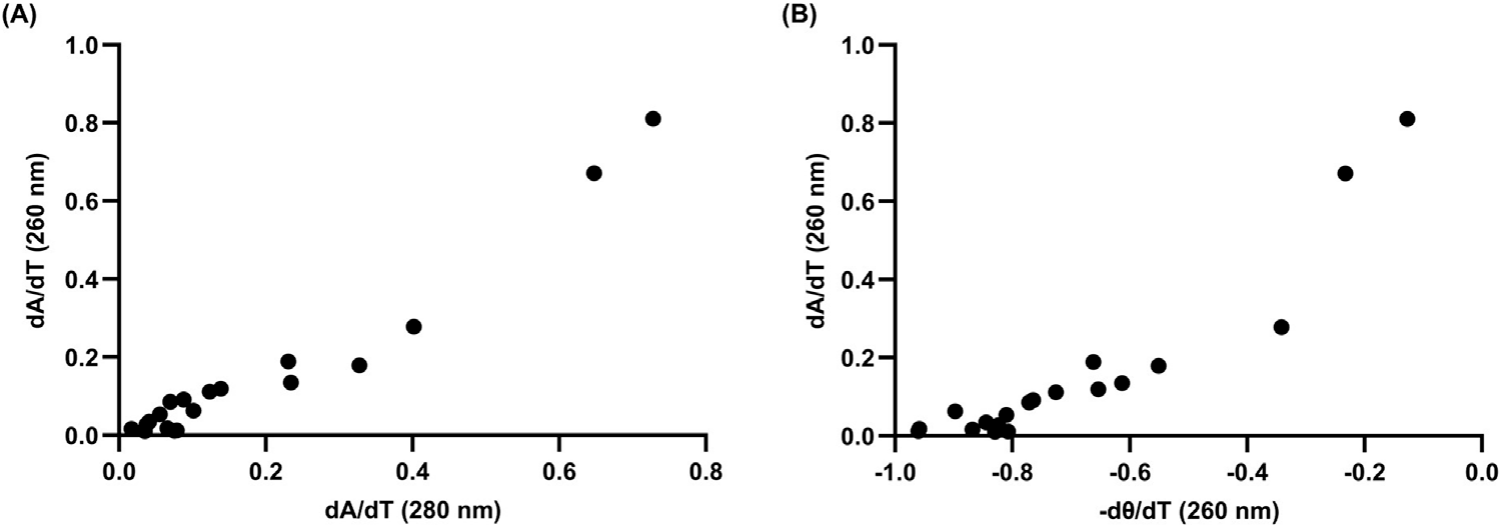
Correlation analyses of 10 µM L15.20.1 under high potassium conditions (50 mM Tris, 150 mM KCl, 1 mM MgCl_2_) to support the use of two-state thermodynamic calculations; n = 3. **(A)** Correlation between hyperchromic wavelengths, 260 nm and 280 nm (r = 0.9702, *p* < 0.0001). **(B)** Correlation at 260 nm between CD and UV-Vis instrumental methods (r = 0.9263, *p* < 0.0001).

There have been a number of approaches to confirm when a two-state model may be accurate in biomolecules using spectrophotometric methods, including identifying correlations between both different methods and different wavelengths (Lumry et al., 1966; Wallimann et al., 2003; Gray and Chaires, 2012). To briefly address the two-state model for a putative rG4 in L15.20.1, melting curve derivatives at 260 nm and 280 nm were used to examine correlations between the wavelengths (Figure 3A). Additionally, two methods (CD and UV-Vis) were compared to one another (Figure 3B). Strong correlations were observed with both approaches. While this alone is likely not sufficient to fully support the application of a two-state model, the visual data is sufficient to afford an overall view of the system and provide enough evidence to support the use of two-state thermodynamic calculations.

### 3.3 Hysteresis

Hysteresis, or shifts in the kinetics of the aptamer during the transition from one state to another (e.g., denaturation to renaturation), may provide details on conformational states and molecularity under specific circumstances, such as changes in temperature or pH (Harkness et al., 2018; Rogers et al., 2018). When hysteresis is not observed during measurement of renaturation, it may indicate that a single, intra-molecular structure exists, rather than that of an inter-molecular structure, which would be kinetically slower in re-forming and thus observable using this method (Hatzakis et al., 2010; Kabbara et al., 2022).

**FIGURE 4:**
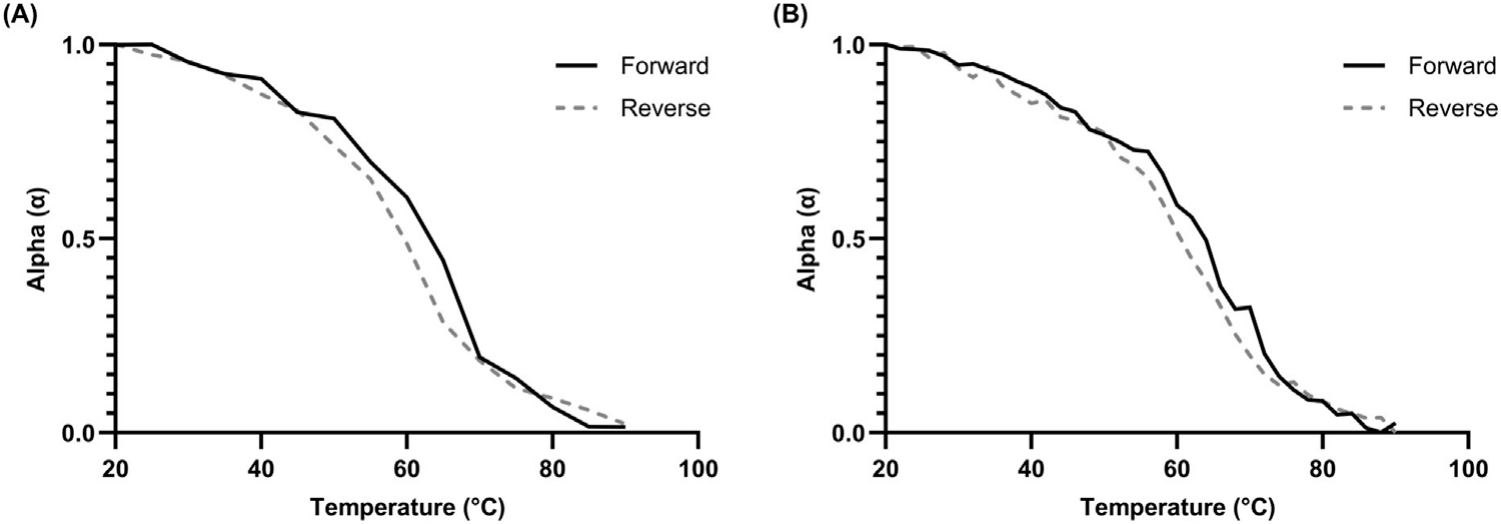
Hysteresis kinetics of 10 µM L15.20.1 at 265 nm under high potassium conditions (50 mM Tris, 150 mM KCl, 1 mM MgCl_2_); n = 3. **(A)** Hysteresis plot with a 3°C/min gradient and 5°C measurement times. **(B)** Hysteresis plot with a 1°C/min gradient and 2°C measurement times.

Hysteresis data was collected for representative L15.20.1. Both fast and slow measurements were performed, and the data was plotted using ellipticity values at 265 nm to examine the changes in folding. (Figure 4). In both approaches, the data indicate that hysteresis is seen within a temperature range of 45°C–70°C but does not exist strongly at other temperatures. This same range of temperatures appears variable in much of the other data collected, including the chromicity plot, and may indicate conformational differences specific to the putative rG4. As more stable structures tend to require more time to fold, and rG4s are particularly stable secondary structures, this shift may be a function of one specific motif. Further, as hysteresis is more often observed in intermolecular structures, it is possible that the rG4 motif is multimolecular in nature, even if the majority of the aptamer may be unimolecular. Overall, these data and that of the derivative and calculated T_m_ values appear sufficient to endorse the application of unimolecular thermodynamic calculations.

### 3.4 Folding kinetics and thermodynamic analysis

Folding and thermodynamic data may also be obtained by employing spectrophotometric methods and calculations, using equations often implemented in protein and nucleic acid analysis (Greenfield, 2006; Hatzakis et al., 2010). Ellipticity values collected throughout temperature-induced denaturation may be utilized to identify folding characteristics and T_m_ values through interpolation and curve fitting. Further, through simple linear regression, the use of van’t Hoff plots of spectrophotometric data can provide free energy (*ΔG*), enthalpy (*ΔH*), and entropy (*ΔS*) values representing the entire system, and individual points within the system, which may be particularly useful in providing information on larger nucleic acids containing multiple motifs within the secondary structure. Moreover, in some cases, molecularity may also be roughly determined through the combination of these and spectrophotometric derivations (Gralla and Crothers, 1973; Marky and Breslauer, 1987; Schroeder and Turner, 2009). Expected thermodynamic values for nucleic acids vary depending upon size, complexity, and stability of the biomolecule. As rG4s are considered highly stable (even more so than dG4s) under the right ionic conditions, aptamers containing them are expected to have higher T_m_ values relative to similarly sized sequences without G4s present, maintenance of overall fold at relatively high temperatures, and favorable thermodynamic values indicative of increased stability. This cation-reliant increase in stability can easily be visualized with ADAM10 under high potassium conditions (Supplementary Figure S1B).

**FIGURE 5:**
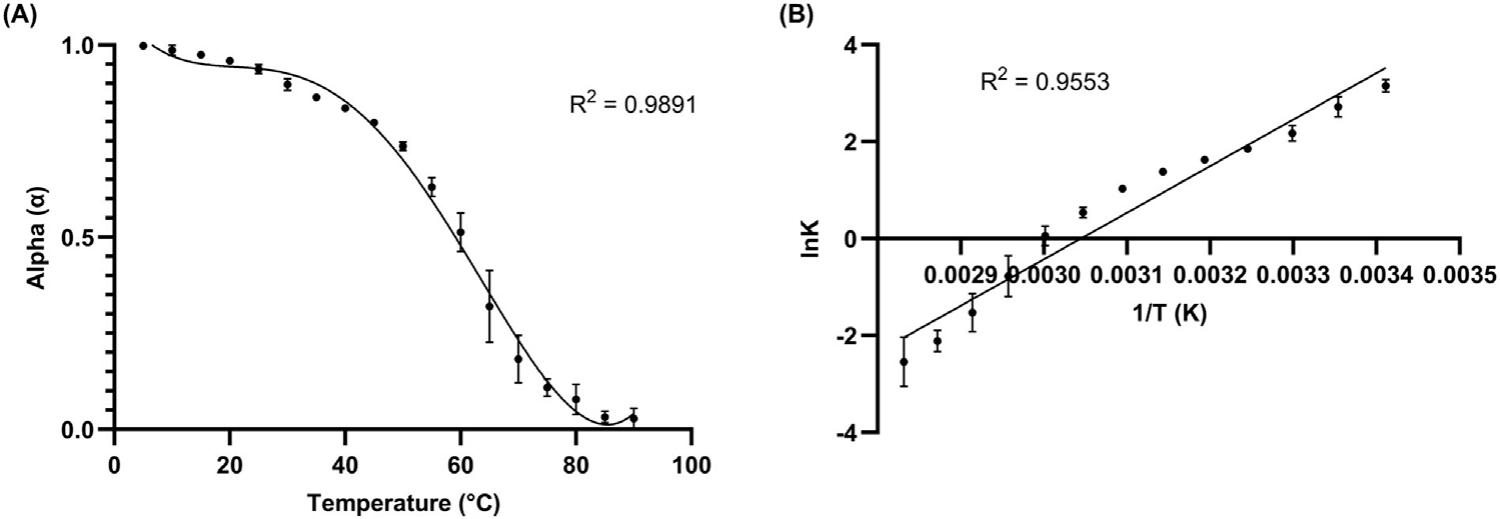
10 µM L15.20.1 under high potassium conditions (50 mM Tris, 150 mM KCl, 1 mM MgCl_2_); n = 3. **(A)** Folding plot providing information on the overall fold and T_m_ value (α = 0.5). **(B)** van’t Hoff plot giving thermodynamic information on the system throughout transition from folded to unfolded state.

Folding and van’t Hoff plots were produced for L15.20.1 (Figure 5; Supplementary Figure S8), and calculated values for L15.20.1 under high potassium conditions fit the above criteria and can be compared to that of high Li^+^ in solution (Supplementary Table S3), which should match values for the aptamer structure under rG4-unfavorable cationic conditions if unimolecular and two-state applicable. The aptamer under rG4-favorable conditions appears characteristic overall for a nucleic acid containing an rG4 compared to one without an rG4 motif, where L15.20.1 under high lithium conditions shows a T_m_ 3°C lower than under high potassium conditions, an overall fold (*α*) decrease of 6%, a *ΔG* that is 24% more positive (indicating decreased ability to form secondary structure spontaneously when compared to the aptamer under high potassium conditions), and an *ΔH* that is 24% more positive (less energetically favorable under high lithium conditions). Further, these plots were produced for the other three aptamers (Supplementary Figures S9–S14), and the data collected for the other representatives also support previous conclusions, where the formation of rG4s or other non-canonical G-related secondary structures in L15.7.1 and H7.10.1 may be possible (Supplementary Table S3). *ΔG*, *ΔH*, *ΔS*, *α*, and T_m_ values indicate increased stability in the three representatives suspected to contain rG4 or other G-rich non-canonical moieties under high potassium conditions compared to high lithium conditions, while representative L15.6.1 shows little variation in any of the values between the two conditions. Moreover, as previous CD data indicate high stability and capacity to refold after high temperature denaturation in all four aptamers (Gruenke et al., 2024), these data further clarify the potential existence of a stable rG4 within L15.20.1, while also supporting some of the conclusions made regarding the other three representatives.

### 3.5 Thioflavin T

ThT has been shown to be a G4-specific fluorophore (Xu et al., 2016; Renaud de la Faverie et al., 2014; Verma et al., 2021) that has found use as a tool for aptasensors (Zheng et al., 2021) and *in vitro* fluorescence assays (Chen and Wang, 2023). It is comprised of a benzothiazole group connected through a rotating C-C bond to a benzeneamine group. ThT produces minimal fluorescence on its own, but when it interacts with certain structures, particularly G4s, the rotation between the two rings can no longer occur, and the resulting hindrance prevents the release of input energy through previously available rotational and vibrational modes, leading to the release of photons to reach ground state. It is theorized that the molecule interacts with nucleic acids containing certain spaces that ThT can occupy, resulting in π- and end-stacking interactions. While ThT can interact with cavities and mismatches (Hanczyc et al., 2021; Lan et al., 2023), its overall fluorescence is much weaker in comparison to the strong signal achieved when complexing with G4 structures.

**FIGURE 6:**
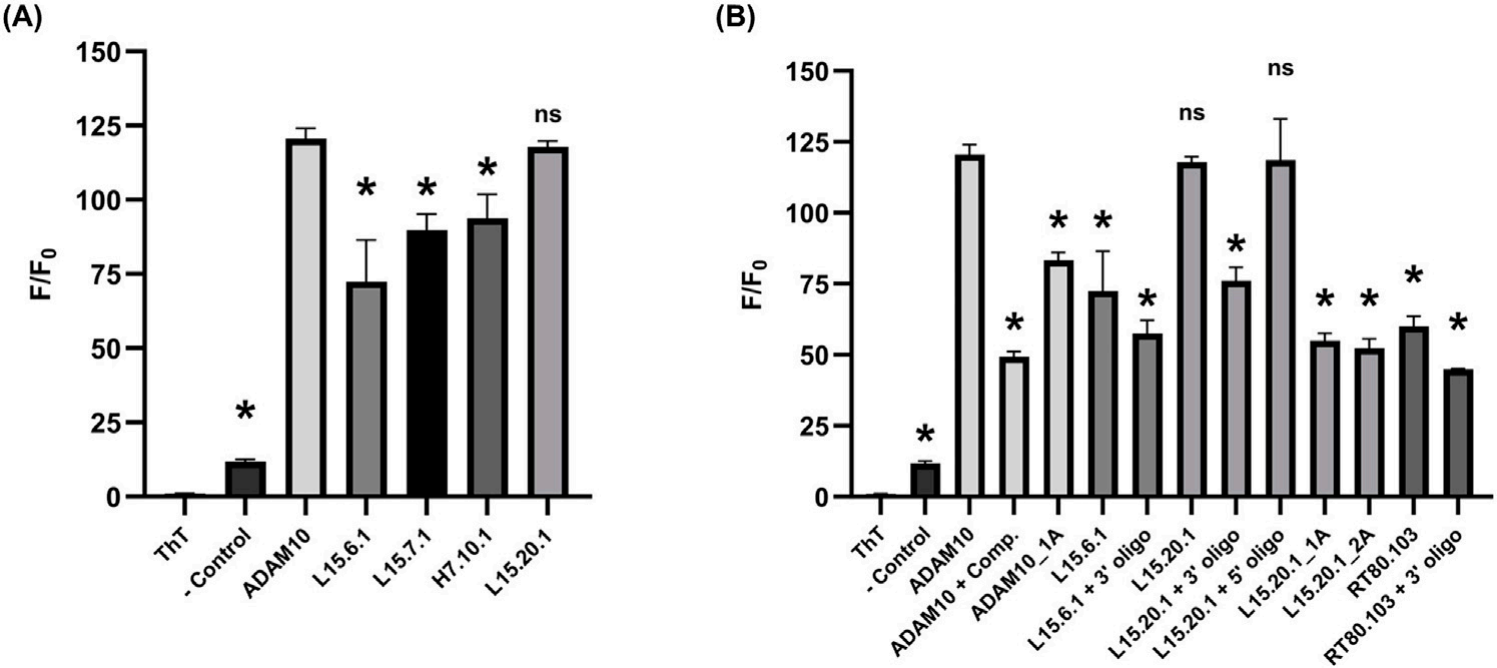
ThT assays under high potassium conditions (50 mM Tris, 150 mM KCl, 1 mM MgCl_2_); n = 3. **(A)** 2 µM:1 µM oligo:ThT of the four aptamer representatives with well volumes of 20 µL. **(B)** 2 µM:1 µM oligo:ThT with annealed complimentary oligos and L15.20.1 mutants for potentially disrupting secondary structures with well volumes of 20 µL. A one-way ANOVA was performed to compare all groups, however, the asterisks indicate statistical significance compared to the ADAM10 positive control (*p*-value <0.0001) and “ns” represents no significance.

Analyses indicate strong ThT-aptamer interactions for L15.20.1, while this is not observed to the same extent for L15.6.1 (Figure 6A). Aptamers L15.7.1 and H7.10.1 appear to vary more in their fluorescence (Supplementary Figure S15), precluding clear determinations. These data further support that rG4 formation may occur in L15.20.1, though not necessarily the potential for rG4 or other G-rich non-canonical structure formation in L15.7.1 or H7.10.1. The data also supports the conclusion that L15.6.1 likely does not contain an rG4, but instead contains only loops and cavities. The prevention of secondary structure folding after denaturation and cooling through the use of annealed complementary sequences provided mostly expected results, where a 3’ oligo annealed to L15.20.1 appears to negatively affect signal intensity in L15.20.1 (Figure 6B). This was anticipated and supports that the 3′constant region of the aptamer likely plays a role in the formation of the non-canonical moiety of interest in L15.20.1. This signal also varies to a significantly larger extent than in L15.6.1, further supporting that potential rG4 formation is disrupted in L15.20.1. As expected, the sequestration of folding at the 5′end did not appear to adversely affect fluorescence signal to a statistically significant degree in L15.20.1. Previous work (Gruenke et al., 2024) demonstrated that G-poor and truncated L15.20.1 mutants demonstrated greatly reduced binding to CA, indicating that the 3′region and the presence of the G-rich motif are necessary for binding. Further, previous endonuclease and TMO structural analyses suggest that the rG4 motif of L15.20.1 likely lies in the 3′region of the aptamer between bases 61 and 84 (Gruenke et al., 2024). Therefore, we also created two mutants to disrupt rG4 formation through G-to-A substitutions in this region (Supplementary Table S1). The first mutant, L15.20.1_1A, replaces a single G with an A in four different G repeats between bases 61 and 84. The second mutant, L15.20.1_2A, replaces 2 Gs in the same four repeats of L15.20.1. Both mutants showed a decrease in fluorescent signal of ∼50% (Figure 6B). Collectively, these and previous binding data (Gruenke et al., 2024) imply that the putative rG4 in L15.20.1 lies within this region of the aptamer and is either necessary for CA binding through direct interaction or may be needed to stabilize other interactions necessary for CA binding. Based on these results, we determined four possible rG4 structures within this base range and had high quality images of each produced (Supplementary Figure S16).

A complimentary oligo annealed to the rG4 positive control, ADAM10 (Xu et al., 2016), resulted in an F/F_0_ signal of ∼50 (a nearly 60% decrease in fluorescence) and could be considered indicative of the signal that would be received by duplexed or cavity-containing nucleic acids. However, to improve our control, we also created an ADAM10 mutant, ADAM10_1A, that interrupts repeats through G-to-A substitutions (Supplementary Table S1). This mutant produced ∼30% less fluorescent signal than the WT. L15.6.1, with a structure predicted to be comprised of duplex, loop, and cavity secondary structures, does not vary much from the complemented ADAM10, and is not overly affected by the prevention of any non-canonical structure formation in the 3′region. In the known RNA bulge control, RT80.103 (Whatley et al., 2013), annealing an oligo to the bulged region to prevent the formation of this motif had little effect. It may be acceptable to determine a minimum signal necessary to warrant G4 investigation with the ThT fluorophore, in which case, an F/F_0_ of ∼60 would be an adequate value to apply to nucleic acids that hold some structure (e.g., cavities or bulges) but do not contain an rG4, and any signal 20 or lower would indicate less-structured RNA or, at the least, structure with far less available ThT-base interaction potential.

This work was also performed utilizing modifications to other methods (Xu et al., 2016; Renaud de la Faverie et al., 2014) and indicate that larger nucleic acids, smaller volumes, and lower concentrations may be used, increasing the range of these assays and reducing the amounts of valuable RNA considered necessary. Variable concentrations and volumes were tested to examine whether smaller amounts of material would produce similar results, and they appeared to be in line with what is seen (Supplementary Figure S15).

## 4 Discussion and conclusion

Overall, this work has allowed for clarification, to a significant extent, as to whether an rG4 moiety exists within the 3′region of L15.20.1, as well as the potential for rG4s or other non-canonical G-rich motifs in two of the other three aptamer representatives. This work has also provided increased confidence that one aptamer, L15.6.1, likely fits its predicted non-rG4 structure, as all data collected indicates this to be the case. Further, some information regarding the molecularity of these aptamers can be gleaned, but care should be taken in data interpretation when examining larger structures with multiple secondary motifs.

Our previous work selecting for and identifying aptamer structural features provided valuable information regarding their ability to bind to and potentially inhibit CA, as well as their amenability to modification for affinity purification of CA from cell lysates (Gruenke et al., 2022; Gruenke et al., 2024). Structural experiments originally focused on predictive, biochemical, and some initial qualitative instrumental analyses, and this work greatly expanded on those data, indicating the existence of non-canonical G-rich secondary structures in three of the four representatives. Biochemical experiments utilizing specific nuclease (T1, S1, and V1) cleaving and alkylating agent (trimethyl oxonium) probing allowed for some structural identification, and when compared with prediction software results, certain regions of each aptamer were well-clarified. However, there were some less defined regions, implying the potential existence of more complex non-canonical structures difficult to identify with these methods. To further clarify and compare data, brief qualitative biophysical analyses using CD spectrophotometry and NMR spectroscopy indicated non-canonical G-rich regions, likely G- or mixed-tetrads, in all but L15.6.1. All four representatives indicated the ability to refold to native conformation after being under lengthy denaturing conditions and were not significantly affected by physiological amounts of Mg^2+^.

Additionally, for L15.20.1, mutations to the putative rG4 G-rich stretches in the 3′region indicate that the rG4 falls between bases 61 and 84, and that changes to this region significantly decrease binding to CA (Gruenke et al., 2024), providing some indications that, should an rG4 exist, it may either be a necessary structure for binding, or may stabilize other interactions required for binding. As rG4s have a number of structure-specific fluorophores, both *in vitro* and *in vivo*, whether or not aptamer conformation changes upon binding would only affect whether we examine “always-on” or “signal-off” fluorescence in future studies. Moreover, as we have the ability to produce these aptamers *in vivo* through plasmid transfection without any *in vitro* steps that could potentially affect aptamer folding, employing approaches such as the use of *in vivo* rG4-specific fluorophores (Xu et al., 2015) to study CA-aptamer interactions using methods such as confocal microscopy, it is possible to confirm the formation of this rG4 motif within cells.

The work presented here centers on spectrophotometric methods and thermodynamic analyses of all four representatives, with a particular focus on L15.20.1. Through the treatment of absorbance, ellipticity, and fluorescence data, as well as the application of thermodynamic and mathematical approaches, additional indications about the overall folds and potential non-canonical secondary structures of each representative were made. Instrumental analyses provided additional support to previous data indicating that L15.6.1 is unimolecular, likely fits its predicted structure, and varies little regardless of cationic environment. The secondary structure of L15.20.1 (under physiological conditions) as a whole may be unimolecular, while the rG4 motif itself may or may not be unimolecular in nature, forming a G- or mixed-quadruplex in the 3′region of the aptamer (which may play a role in CA binding). L15.7.1 may contain a G-rich non-canonical structure, is reliant on cationic environment, and has indeterminant molecularity. H7.10.1 is the most ambiguous of the representatives, but almost certainly contains an elusive G-rich non-canonical structure that may potentially undergo structure-switching in a temperature- or ion-dependent manner. It is likely highly polymorphic, particularly dependent on cationic conditions, and does not meet the criteria for a unimolecular system. Instead, it is more likely multimolecular and contains a number of significant donating conformations.

The combination of these approaches also provided a cost- and time-effective way to determine energetic values throughout the denaturation-renaturation process. Some general results were expected for these aptamers, as they have already been shown to refold after temperature-based denaturation, and mostly allow for accurate folding and thermodynamic calculations under high salt conditions. All representatives appear enthalpically/energetically (but not entropically) favorable, with spontaneous folding at lower temperatures. If calculated correctly, at the T_m_ value for each aptamer, Gibbs free energy (*ΔG*) should reach zero, while the reaction quotient (*Q*) and folding constant (*K*) should reach equilibrium (*Q/K* = 1.0). This is what was seen for all thermodynamic experiments and calculations performed on the four representatives. Further, variations in *α*, *ΔG*, *ΔH*, *ΔS*, and *Q/K* were calculated for each aptamer throughout folding, providing some insight into potential structural changes at any given temperature, and can be seen in Supplementary Table S4 for L15.20.1. This is a key feature of the data collected, as although nucleic acids are often treated *via* a two-state model, this is not always applicable and may be less so when examining extremely stable nucleic acids (like ADAM10 under high potassium conditions) or fairly large oligonucleotides with a high number of secondary architectures. Thus, examining variations throughout denaturation may provide some insight into the number of potential states and structures.

The thermodynamic and kinetic information gathered for L15.20.1 has allowed for speculation regarding the behavior of the aptamer. These data all appear to signal variability within the range of 45°C–70°C, and there are indications that at least two major temperature-dependent forms of the aptamer exist and dominate spectra. Enthalpy data appear to generally imply two states throughout melting, where a more positive trend ceases as the aptamer approaches 45°C (Supplementary Table S4). The chromicity plot may imply that the major form of the aptamer below this range contains an rG4 motif which begins to melt near 45°C, however, hyperchromicity is visible after 70°C, which may be indicative of a different major form melting. Similarly, hysteresis only appears pronounced within this range, indicating the potential for both unimolecular and multimolecular temperature-dependent structures. The data was determined to fit well at different wavelengths and with different methods, and the melting temperatures were confirmed to be similar through calculation and derivation approaches, indicating the overall applicability of a two-state model and potential molecularity information. However, the variability within the range of 45°C–70°C does not necessarily fit with this conclusion.

One possibility is that the aptamer is primarily unimolecular in nature, but the region of the rG4 may instead be multimolecular. It is also possible that multimeric interfaces of G-tetrads could play a role in why these data fit well, where tetrads from different strands stack, rather than forming between multiple strands (Kolesnikova and Curtis, 2019). These data would also fit with what is known about parallel, bimolecular G4s, which often have multiple two G-stretches, short loop lengths, and rely more heavily on potassium (Mullen et al., 2012; Tang and Shafer, 2006; Puig Lombardi and Londoño-Vallejo, 2019; Hazel et al., 2004). Further, this data is important when considering applications and conditions for aptamer use, and these types of experiments may be beneficial for those intending to employ aptamers under conditions varying from those used during the selection process.

An aptamer such as L15.20.1, that functions under physiological conditions, forms a fluorophore-specific structure, and is not plagued by self-aggregation, might allow us to more easily study aptamer-CA binding and interactions. It may also have the potential for instrumental analyses within cells, providing an opportunity to examine these relationships *in vivo* that may otherwise be impossible due to modification interference. Further, this aptamer may be employed to study different aspects of viral replication and determine what roles CA may play throughout these processes.

## Data Availability

The original contributions presented in the study are included in the article/Supplementary Material, further inquiries can be directed to the corresponding author.
