# Supplementary material for: G-quadruplex formation in RNA aptamers selected for binding to HIV-1 capsid
Source: Front Chem. 2024 Oct 22;12:1425515. doi: 10.3389/fchem.2024.1425515 (PMC11536715; doi:10.3389/fchem.2024.1425515)
Supplement: Supplementary file 1 [file Presentation1.pdf]

Supplementary Material

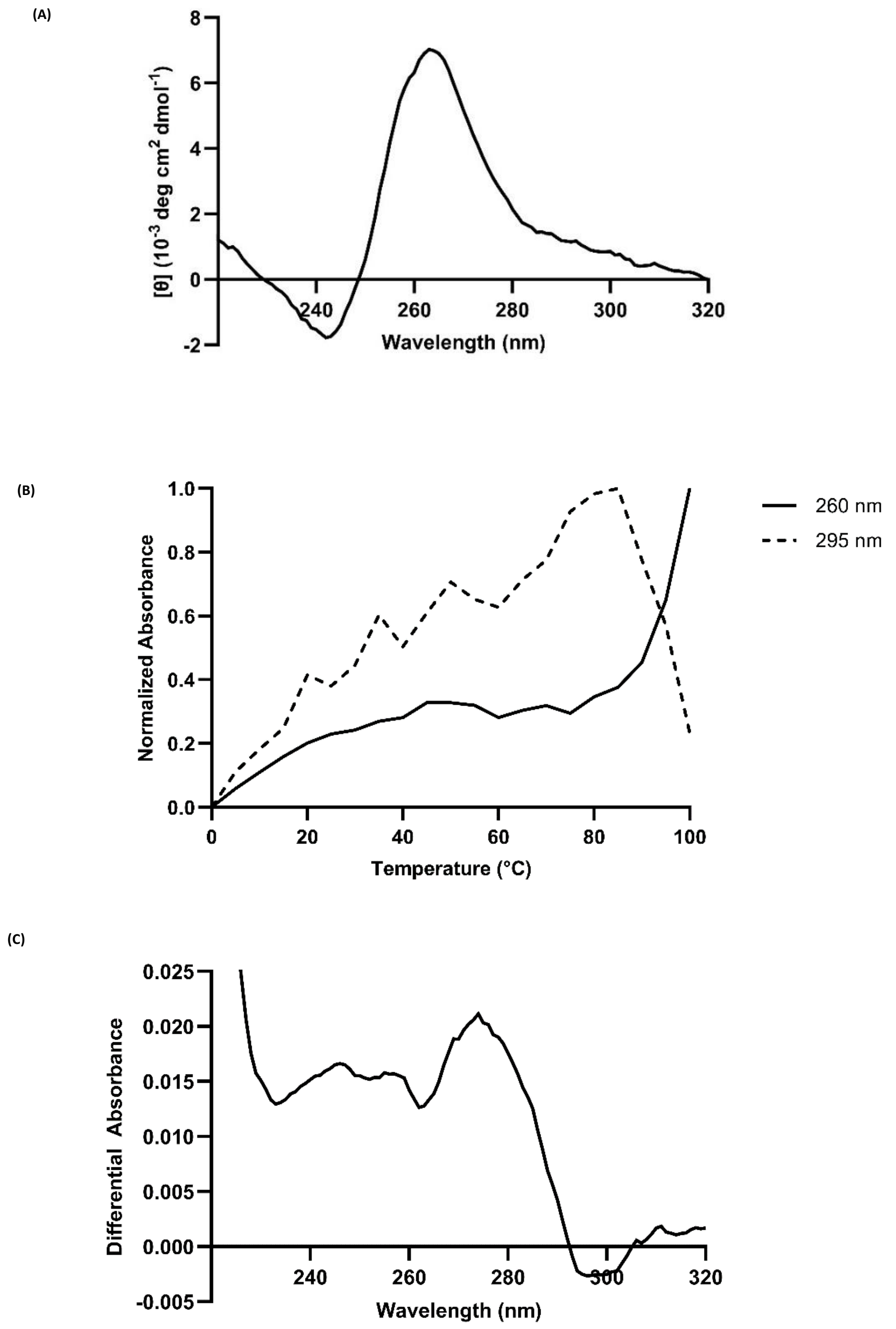

Supplementary Figure 1: 10 µM ADAM10 in 50 mM Tris, 150 mM KCl, and 1 mM $MgCl_2$. **A)** CD spectrum. **B)** Chromicity plot at 260 nm and 295 nm. **C)** TDS.

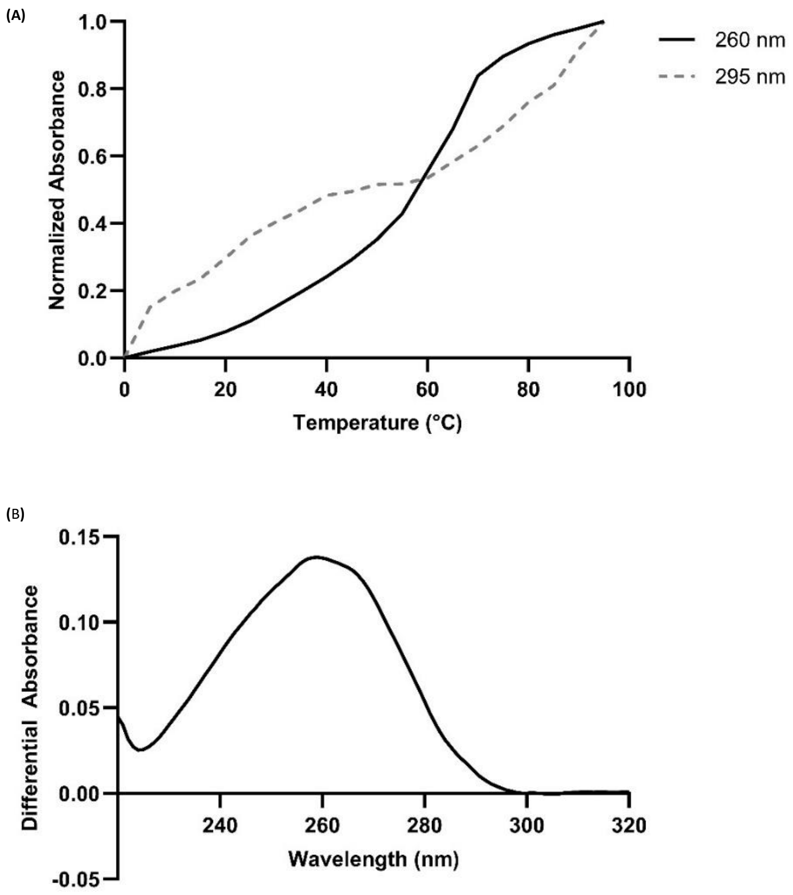

Supplementary Figure 2: 10 µM L15.20.1 under high lithium conditions (50 mM Tris, 150 mM LiCl, 1 mM $MgCl_2$); n = 3. **A)** Chromicity plot at 260 nm and 295 nm. **B)** TDS of L15.20.1.

(A)

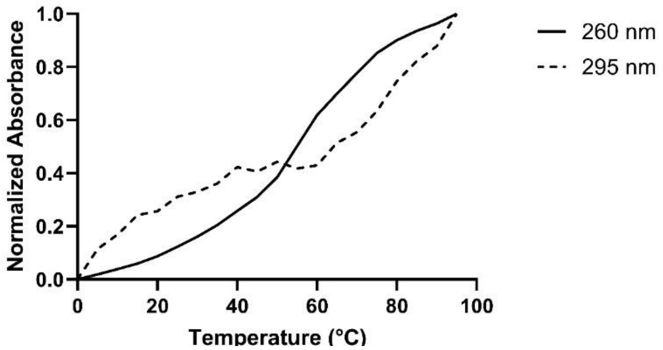

(B)

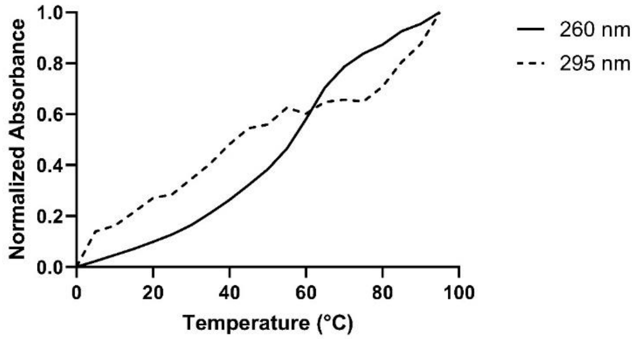

(C)

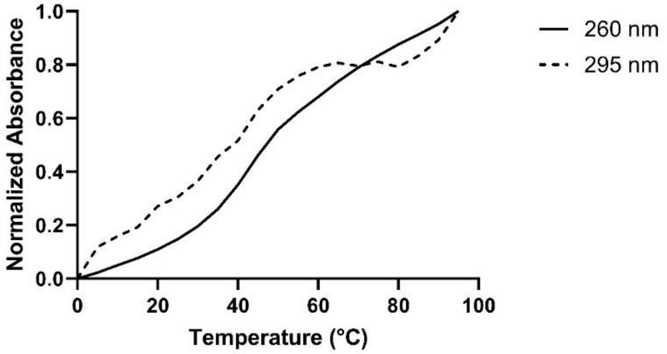

Supplementary Figure 3: 10 µM aptamers under high potassium conditions (50 mM Tris, 150 mM KCl, 1 mM $MgCl_2$). Chromicity plots indicate no hypochromism in **A)** L15.6.1, **B)** L15.7.1, **C)** H7.10.1.

(A)

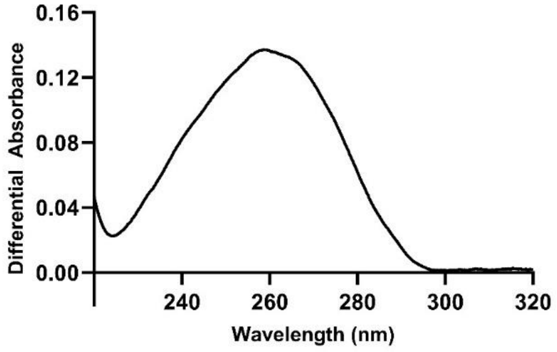

(B)

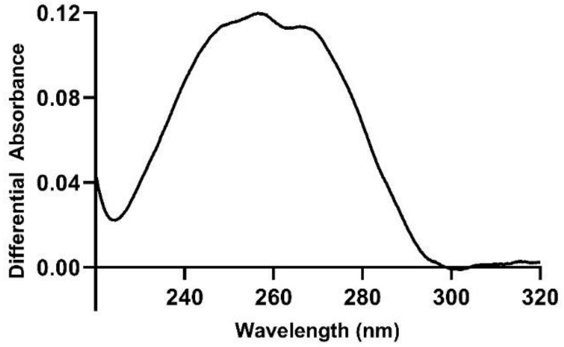

(C)

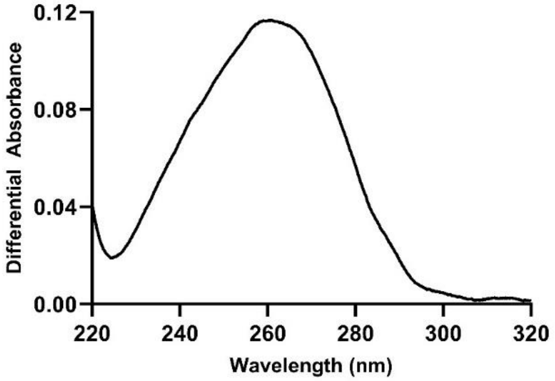

Supplementary Figure 4: 10 µM aptamers under high potassium conditions (50 mM Tris, 150 mM KCl, 1 mM $MgCl_2$). TDS plots indicate no hypochromism in **A)** L15.6.1, **B)** L15.7.1, **C)** H7.10.1.

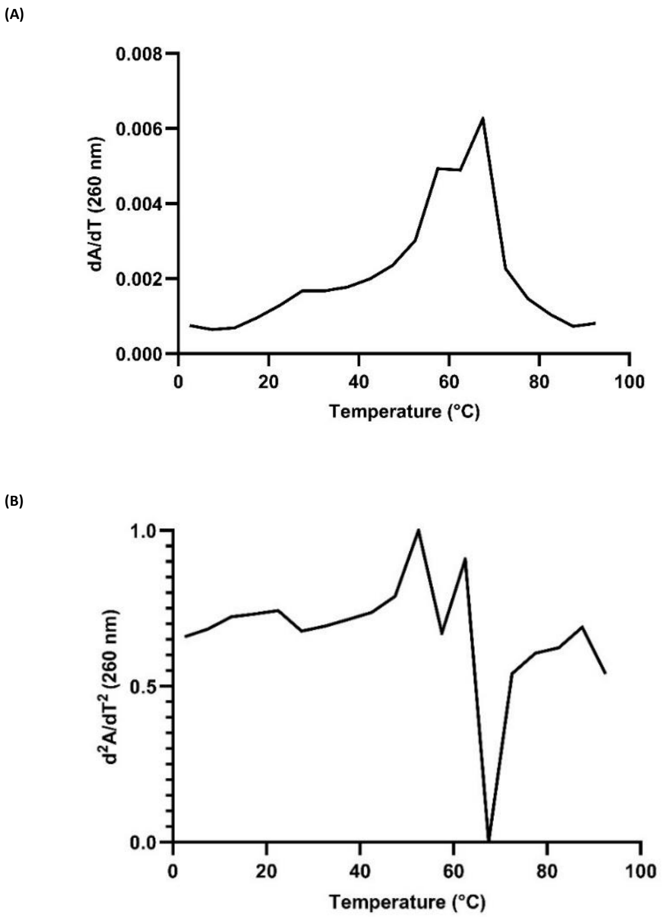

Supplementary Figure 5: 10 µM L15.20.1 under high lithium conditions (50 mM Tris, 150 mM LiCl, 1 mM $MgCl_2$); n = 3. **A)** Absorbance derivative plot with peak spanning 57.5°C to 67.5°C. **B)** Absorbance second derivative plot at 260 nm clarifying the derived T_m of 67.5°C at y = 0. **C)** The calculated values of the system.

(A)

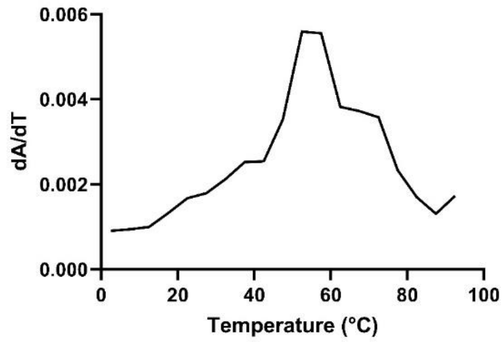

(B)

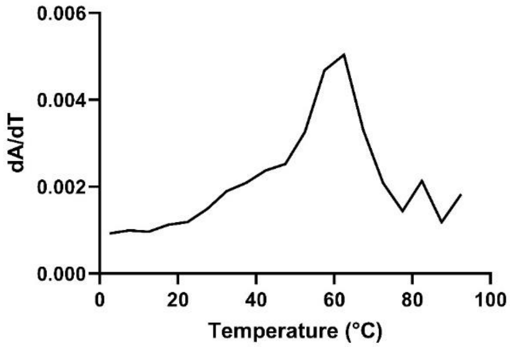

(C)

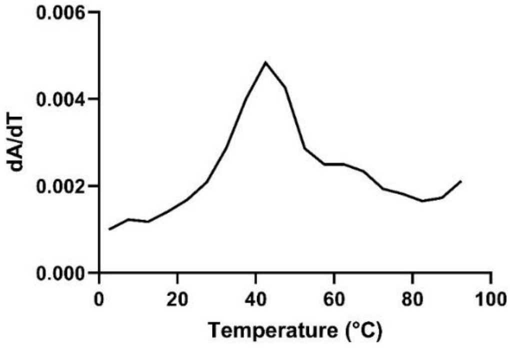

Supplementary Figure 6: First derivative plots at 260 nm of 10 µM aptamers under high potassium conditions (50 mM Tris, 150 mM KCl, 1 mM $MgCl_2$). **A)** L15.6.1, **B)** L15.7.1, and **C)** H7.10.1**.**

(A)

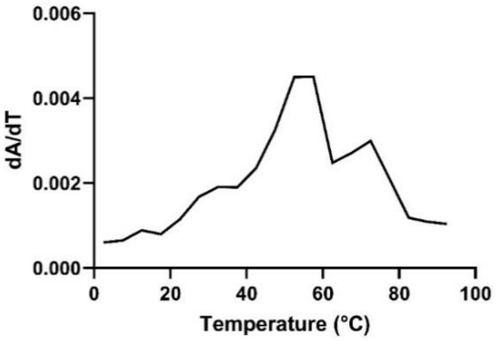

(B)

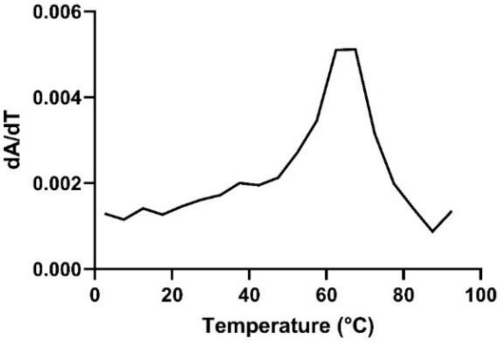

(C)

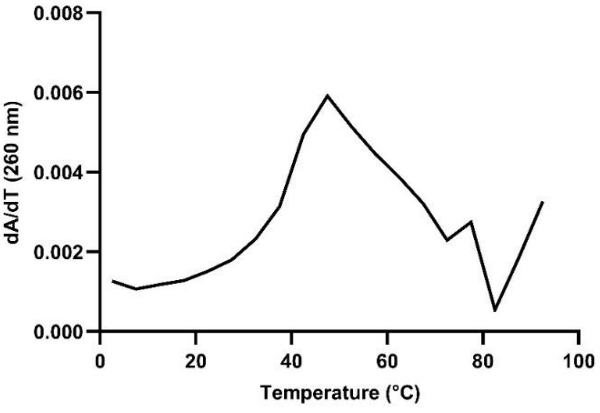

Supplementary Figure 7: First derivative plots of 10 µM aptamers at 260 nm under high lithium conditions (50 mM Tris, 150 mM KCl, 1 mM $MgCl_2$). **A)** L15.6.1, **B)** L15.7.1, and **C)** H7.10.1**.**

(A)

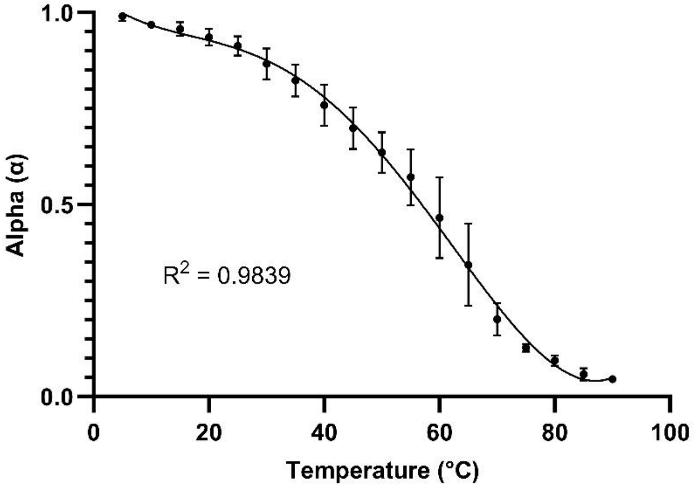

(B)

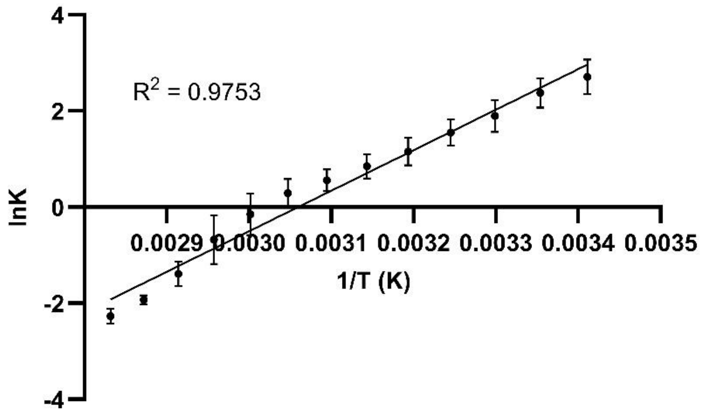

Supplementary Figure 8: 10 µM L15.20.1 under high lithium conditions (50 mM Tris, 150 mM LiCl, 1 mM $MgCl_2$); n = 3. **A)** Folding (α) plot providing information on the overall fold and T_m value (α = 0.5). **B)** van 't Hoff plot giving thermodynamic information on the system throughout transition from folded to unfolded state.

(A)

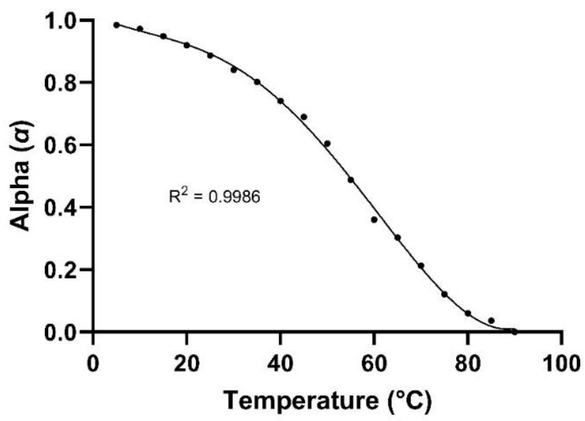

(B)

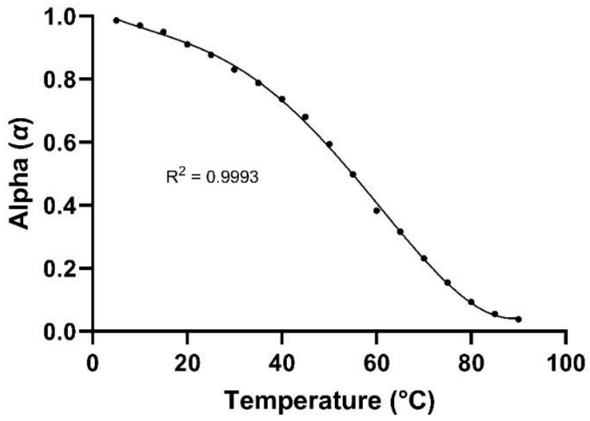

Supplementary Figure 9: Folding plots for 10 µM L15.6.1 under **A)** high potassium conditions (50 mM Tris, 150 mM KCl, 1 mM $MgCl_2$) and **B)** high lithium conditions (50 mM Tris, 150 mM LiCl, 1 mM $MgCl_2$).

(A)

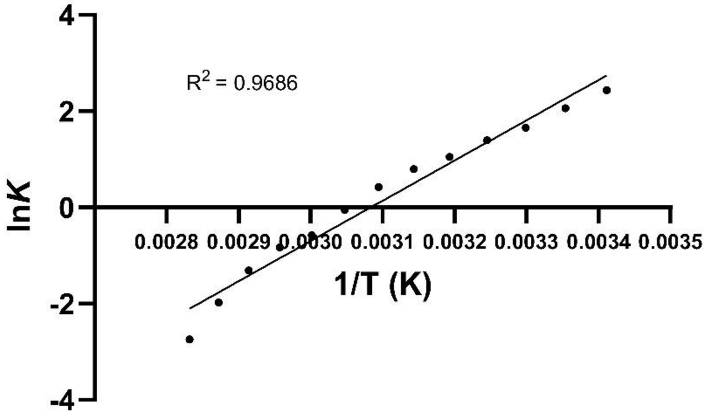

(B)

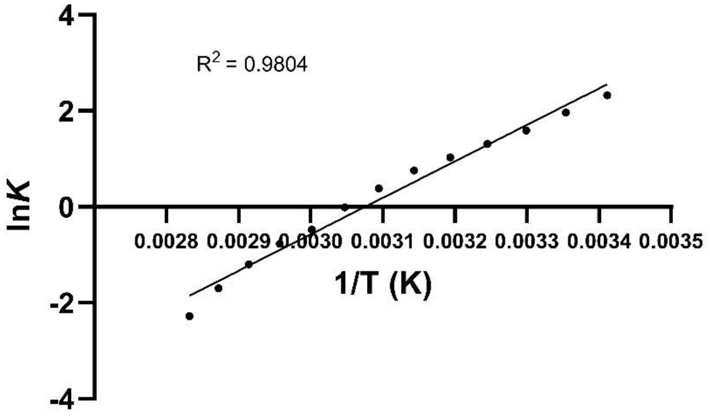

Supplementary Figure 10: van 't Hoff plots for 10 µM L15.6.1 under **A)** high potassium conditions (50 mM Tris, 150 mM KCl, 1 mM $MgCl_2$) and **B)** high lithium conditions (50 mM Tris, 150 mM LiCl, 1 mM $MgCl_2$).

(A)

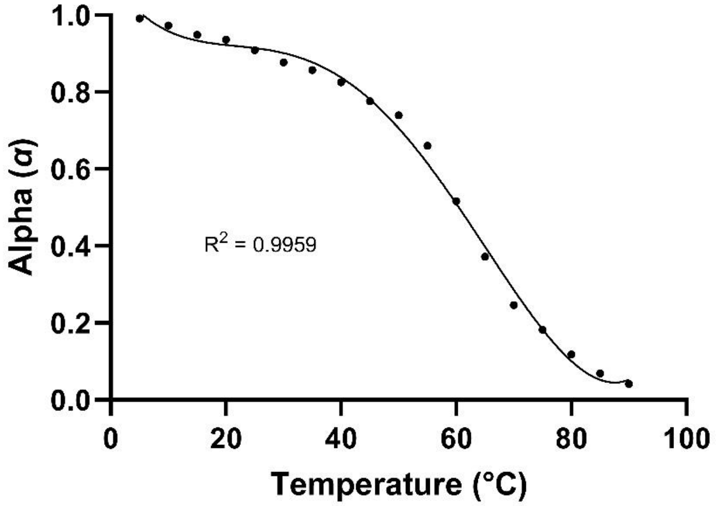

(B)

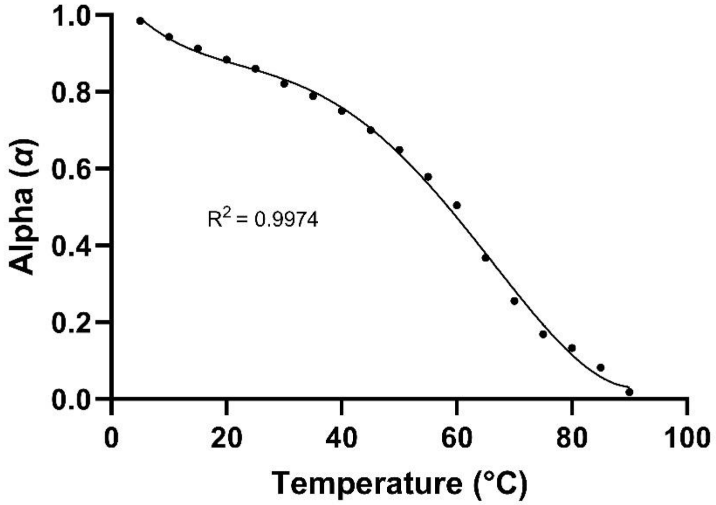

Supplementary Figure 11: Folding plots for 10 µM L15.7.1 under **A)** high potassium conditions (50 mM Tris, 150 mM KCl, 1 mM $MgCl_2$) and **B)** high lithium conditions (50 mM Tris, 150 mM LiCl, 1 mM $MgCl_2$).

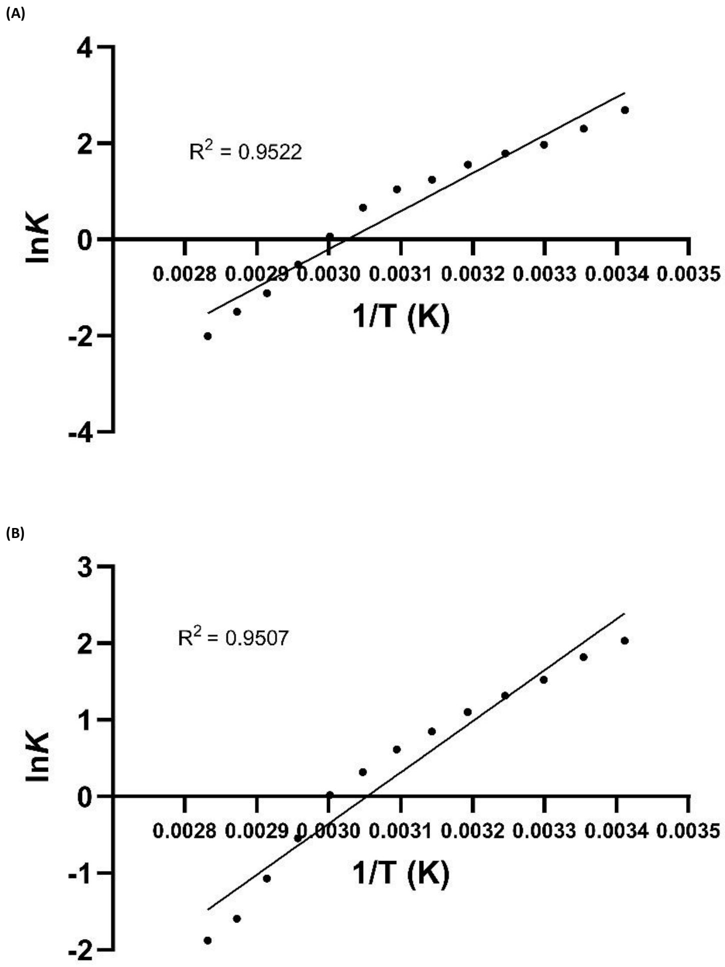

Supplementary Figure 12: van ‘t Hoff plots for 10 µM L15.7.1 under **A)** high potassium conditions (50 mM Tris, 150 mM KCl, 1 mM $MgCl_2$) and **B)** high lithium conditions (50 mM Tris, 150 mM LiCl, 1 mM $MgCl_2$).

(A)

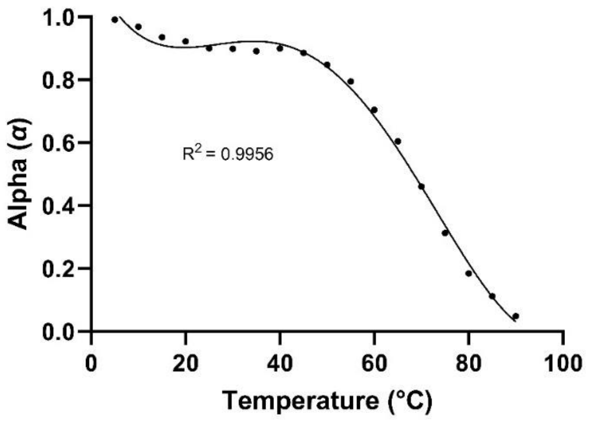

(B)

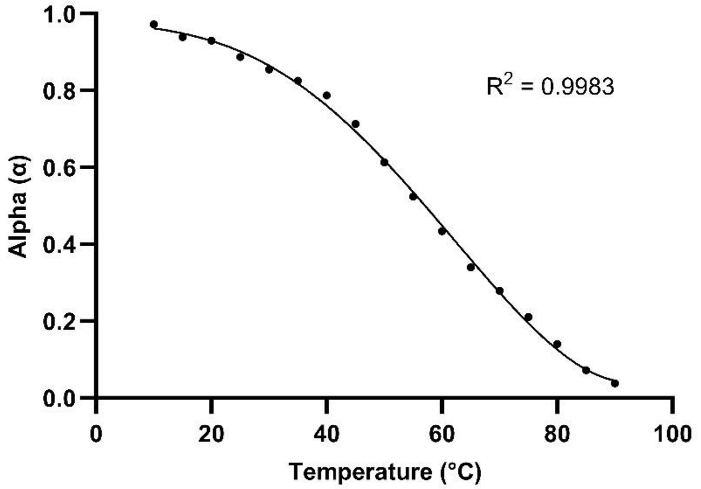

Supplementary Figure 13: Folding plots for 10 µM H7.10.1 under **A)** high potassium conditions (50 mM Tris, 150 mM KCl, 1 mM $MgCl_2$) and **B)** high lithium conditions (50 mM Tris, 150 mM LiCl, 1 mM $MgCl_2$).

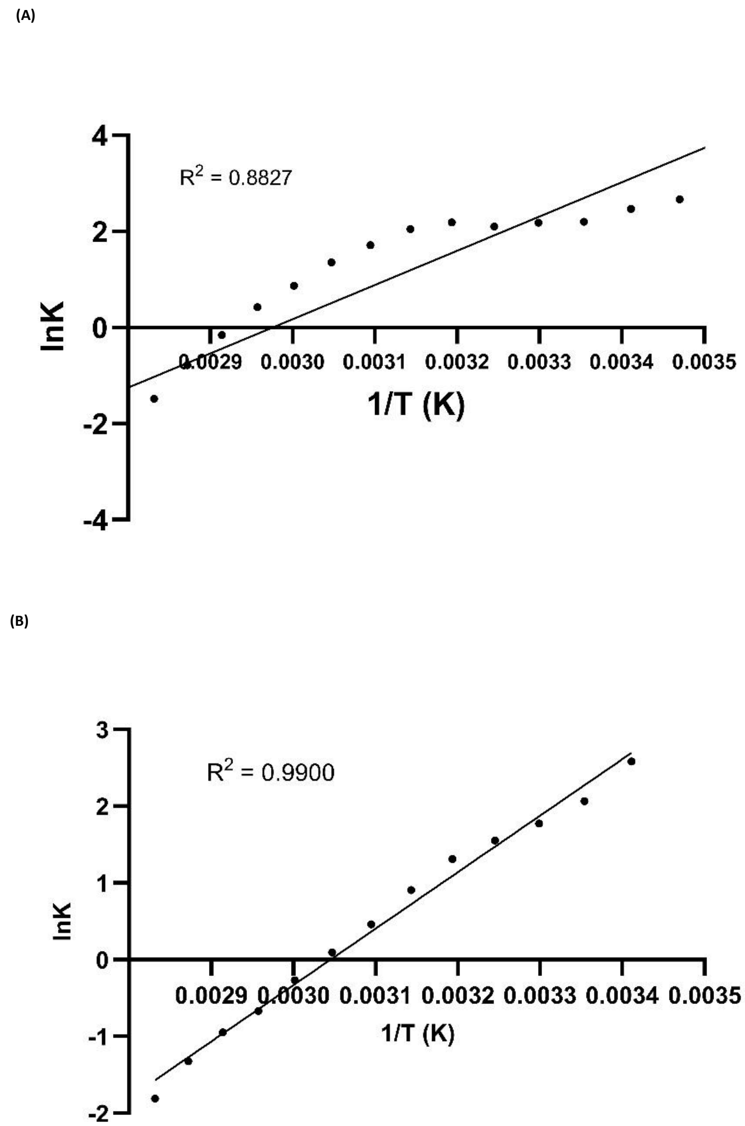

Supplementary Figure 14: van 't Hoff plots for 10 μM H7.10.1 under **A)** high potassium conditions (50 mM Tris, 150 mM KCl, 1 mM $MgCl_2$) and **B)** high lithium conditions (50 mM Tris, 150 mM LiCl, 1 mM $MgCl_2$).

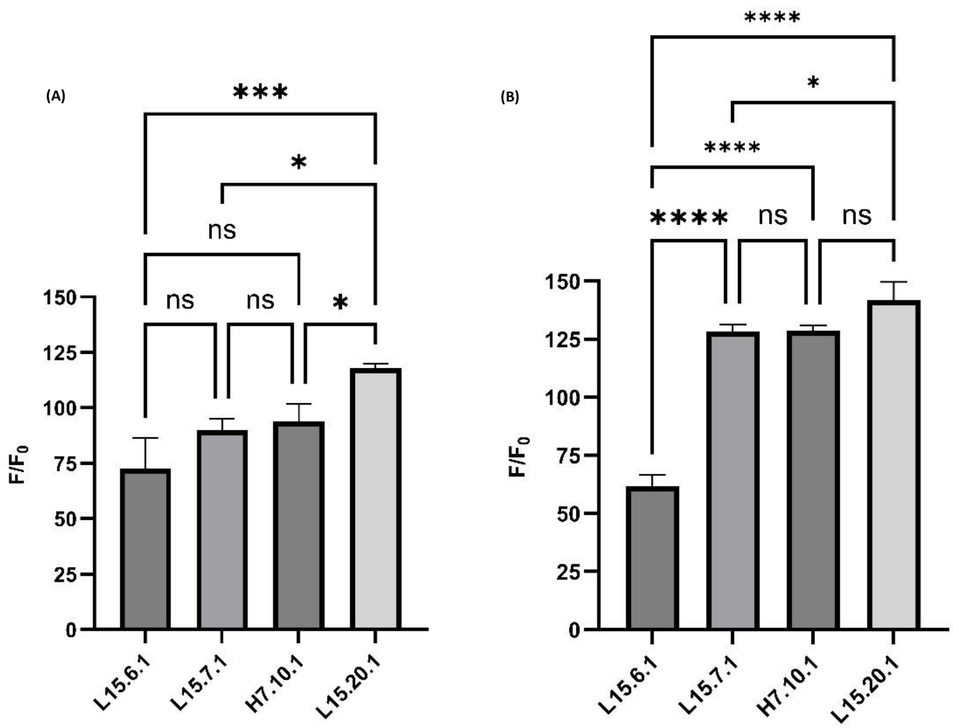

Supplementary Figure 15: ThT assay under high potassium conditions (50 mM Tris, 150 mM KCl, 1 mM $MgCl_2$); n = 3. 2 µM:1 µM oligo:ThT of the four aptamer representatives with well volumes of 20 µL. **A)** Displays a one-way ANOVA performed to measure significance between aptamers for the data in Figure 6A. **B)** A second set of data collected where aptamers L15.7.1 and H7.10.1 appeared to vary significantly from the first. Again, a one-way ANOVA was performed to display the significance between each of the four aptamers (ns = not significant and asterisks (*) indicate significance).

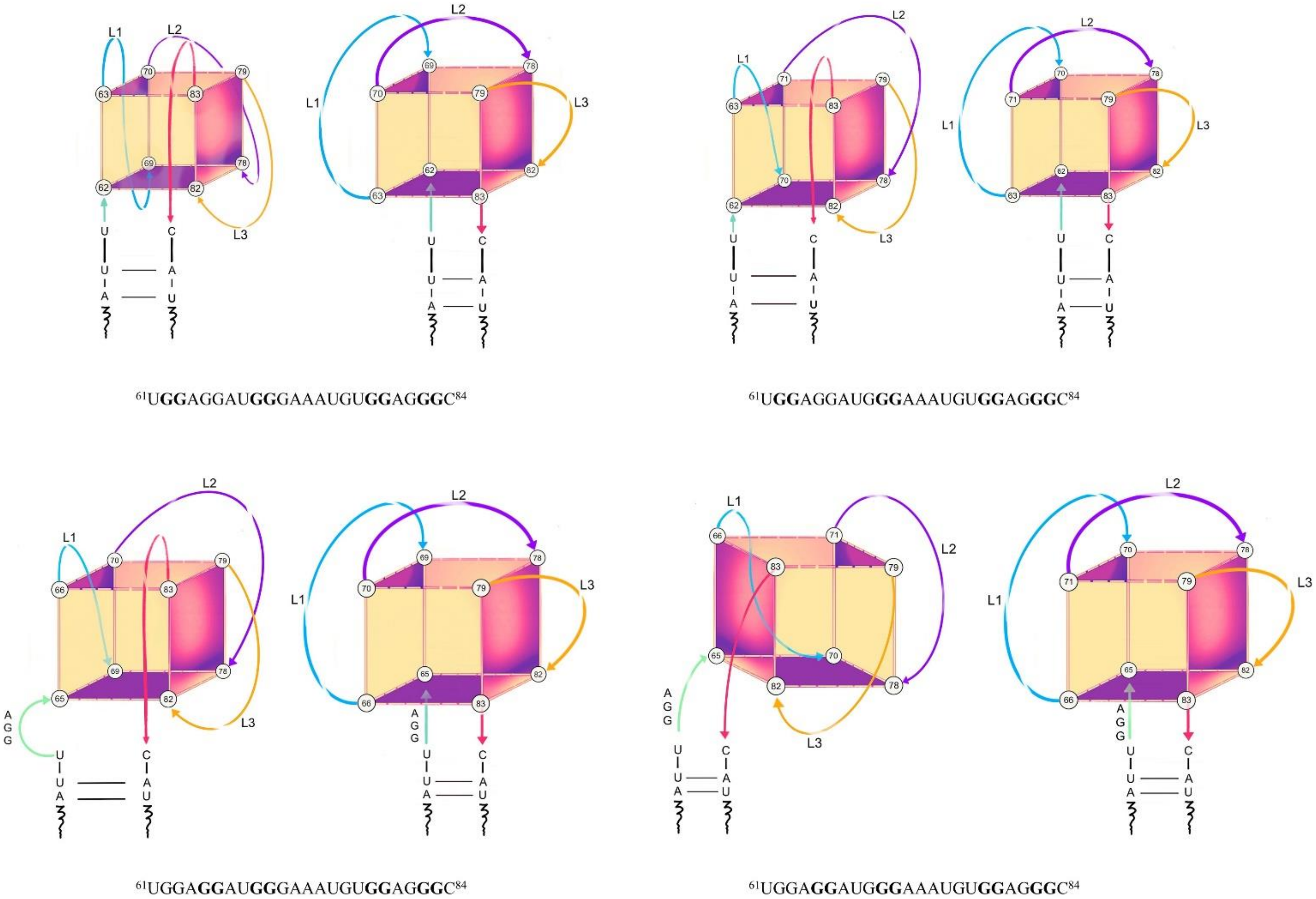

Supplementary Figure 16: Potential rG4 motifs within aptamer L15.20.1 based on endonuclease and TMO data indicating the region from base 61 to base 84 to be the likely region of the rG4 structure. Images produced by artist Miranda Tess Knutson.

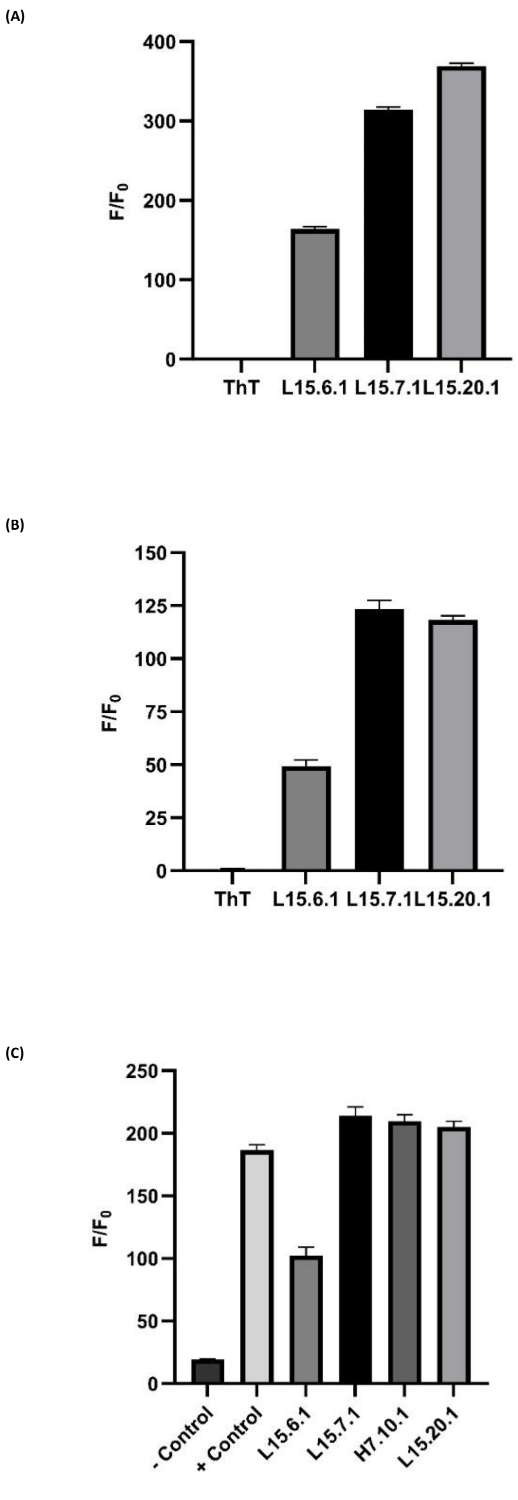

Supplementary Figure 17: Varying volumes and concentrations of RNA and ThT for 96-well plate fluorescence assays under high potassium conditions (50 mM Tris, 150 mM KCl, 1 mM $MgCl_2$); n = 3. **A)** Assays performed using 4 µM:2 µM oligo:ThT with well volumes of 100 µL. **B)** Assays performed using 1 µM:0.5 µM oligo:ThT with well volumes of 100 µL. **C)** Assays performed using 4 µM:2 µM oligo:ThT with well volumes of 40 µL.

Supplementary Table 1: Aptamer, control, mutant, primer, and complementary oligo sequences. Bold G's in the L15.20.1 sequence suspected to potentially play a role in rG4 formation. Bold and underlined bases in other sequences indicate mutations made for the disruption of rG4 formation.

Aptamers and Oligos	Sequences (5'→3')
L15.6.1	GGAAGAAGAGAAUCAUACACAAGAUCGACGUACCUCAGGGUGGUGUAUGACUGAGGUGAAGACUGUGAACCAUGGCAUGCGGGCAUAAGGUAGGUAAGUCCAUA
L15.6.1 3' Oligo	TATGGACTTACCTACCTTATGCCC
L15.7.1	GGAAGAAGAGAAUCAUACACAAGACCAACCAGAGACGCACCAGAGUCCUCGAGGGAAAGGAGUGGGUGGUACGGGACAUGGGGCAUAAGGUAGGUAAGUCCAUA
H7.10.1	GGAAGAAGAGAAUCAUACACAAGAUGGCGGGUAAACUAAGUCCUUGGGAGGGAGCGAUGGUUCUUAGCCUCGAGGGAUGGGGGCAUAAGGUAGGUAAGUCCAUA
L15.20.1	GGAAGAAGAGAAUCAUACACAAGAGGGUCGCAUCGGUCUCUAUGGAUAAAUCUGCCUUAUU**GG**A**GG**AU**GGG**AAAUGU**GG**AG**GG**CAUAAGGUAGGUAAGUCCAUA
L15.20.1 3' Oligo	TATGGACTTACCTACCTTATGCCC
L15.20.1 5' Oligo	GGAAGAAGAGAATCATACACAAGA
L15.20.1_1A - 1	GGAAGAAGAGAATCATACACAAGAG
L15.20.1_1A - 2	CTCTATGGATAAATCTGCCTTATTGAAGGATGAGAAATG
L15.20.1_1A - 3	TTCTCTTAGTATGTGTTCTCCCAGCGTAGCCAGAGATACCTATTTAG
L15.20.1_1A - 4	CTTCCTACTCTTTACACTTCTCGTATTCCATCCATTCAGGTAT
L15.20.1_2A - 1	GGAAGAAGAGAATCATACACAAGAG
L15.20.1_2A - 2	CTCTATGGATAAATCTGCCTTATTAAAGGATAAGAAATG
L15.20.1_2A - 3	TTCTCTTAGTATGTGTTCTCCCAGCGTAGCCAGAGATACCTATTTAG
L15.20.1_2A - 4	TTTCCTATTCTTTACATTTCTTGTATTCCATCCATTCAGGTAT
L15.20.1_1A Mutant	GGAAGAAGAGAAUCAUACACAAGAGGGUCGCAUCGGUCUCUAUGGAUAAAUCUGCCUUAUUG**A**AGGAUG**A**GAAAUGUG**A**AG**A**GCAUAAGGUAGGUAAGUCCAUA
L15.20.1_2A Mutant	GGAAGAAGAGAAUCAUACACAAGAGGGUCGCAUCGGUCUCUAUGGAUAAAUCUGCCUUAUU**AA**AGGAU**AA**GAAAUGU**AA**AG**AA**CAUAAGGUAGGUAAGUCCAUA
RT80.103	GGGCAUAAGGUAUUUAAUUCCAUAGCAACCGGUUGUCUACACGCGGCGAAUAGAGCCCGGUUCAAGGACACCGCCACUGCUGUCGACAUUUCCUUAGUGCUAGAUUGAUUCGGAUGCU CCGGUAGCUCAACCUG
RT80.103 3' Oligo	GGTGTCCTTGAACCG
ADAM10 (+ Control)	GGGGGACGGGUAGGGGCGGGAGGUAGGGG
ADAM10 Compliment	CCCCTACCTCCCGCCCCTACCCGTCC
ADAM10_1A Mutant	G**A**G**A**GACG**A**GUA**A**G**A**GCG**A**GA**A**GUA**A**G**A**G
MJL435 (- Control)	GAUUGGCGCAUGUGUCAAUGCUUCCUUUGCCAAAUAAUCCGCAGAAUC

Supplementary Table 2: Calculated values for the L15.20.1 aptamer system under either high potassium or high lithium conditions (50 mM Tris, 150 mM XCl (X = K^+ or Li^+), 1 mM $MgCl_2$.).

**L15.20.1**			
High Potassium	**System**		**Error**
$T_m$avg	59.89	+/-	1.83
α_{avg}	0.856	+/-	0.006
ΔH_{system} (kcal/mol)	-19.79	+/-	1.31
ΔS_{system} (cal K/mol)	-60.49	+/-	4.37
High Lithium			
$T_m$avg	57.56	+/-	4.02
α_{avg}	0.801	+/-	0.039
ΔH_{system} (kcal/mol)	-16.05	+/-	0.05
ΔS_{system} (cal K/mol)	-49.49	+/-	0.16

Supplementary Table 3: Table representing overall probability of an rG4 existing within the four aptamer representatives at 10 µM under high lithium and high potassium conditions (50 mM Tris, 150 mM XCl (X = K^+ or Li^+), and 1 mM $MgCl_2$).

		$Tm_{\alpha = 0.5}$ (°C)	dA/dT (260 nm)	$\alpha_{37°C}$	$\Delta G_{37°C}$ (kcal/mol)	ΔH (kcal/mol)	ΔS (cal/K/mol)	Denatured/Refold	Mg^{2+} Effects	^1H Imino Signal	Hypochromicity (295 nm)	G4
L15.6.1	Li+	55	55	0.77	-0.75	-11.95	-36.14	+	-	-	-	-
	K+	55	55	0.78	-0.79	-14.89	-45.47	+			-	
L15.7.1	Li+	60	65	0.77	-0.74	-7.14	-20.63	+	-	+	-	~
	K+	61	62.5	0.84	-1.03	-7.39	-20.53	+			-	
H7.10.1	Li+	56.5	47.5	0.8	-0.86	-6.22	-17.28	+	-	+	-	~
	K+	68	42.5	0.91	-1.42	-8.1	-21.52	~			-	
L15.20.1	Li+	57.5	67.5	0.8	-0.86	-16.05	-49.49	+	~	+	-	+
	K+	60	61	0.86	-1.13	-21.02	-63.31	+			+	

Supplementary Table 4: 10 µM L15.20.1 under high potassium conditions (50 mM Tris, 150 mM KCl, 1 mM $MgCl_2$); n = 3. Thermodynamic properties for the system throughout the unfolding process.

°C	Kelvin	K_{avg}	+/-	lnK_{avg}	+/-	ΔG_{avg} (kcal/mol)	+/-	ΔH_{avg} (kcal/mol)	+/-	Q/K_{avg}	+/-	ΔS_{avg} (cal K/mol)	+/-
20	293.15	23.50	2.85	3.15	0.13	-1.83	0.07	-17.33	16.25	0.04	0.01	-59.10	55.42
25	298.15	15.39	3.26	2.72	0.21	-1.61	0.12	-14.98	4.33	0.07	0.01	-50.24	14.52
30	303.15	8.85	1.41	2.17	0.16	-1.30	0.10	-19.58	6.99	0.11	0.02	-64.59	23.07
35	308.15	6.37	0.42	1.85	0.07	-1.13	0.04	-11.93	4.80	0.16	0.01	-38.71	15.58
37	310.15	5.97	0.29	1.79	0.05	-1.10	0.03	-10.30	1.90	0.17	0.01	-29.66	6.04
40	313.15	5.08	0.10	1.62	0.02	-1.01	0.01	-8.59	1.92	0.20	0.00	-27.41	6.13
45	318.15	3.96	0.22	1.38	0.06	-0.87	0.03	-9.80	1.87	0.25	0.01	-30.81	5.88
50	323.15	2.80	0.16	1.03	0.06	-0.66	0.04	-14.20	3.82	0.36	0.02	-43.93	11.83
55	328.15	1.71	0.19	0.53	0.11	-0.35	0.07	-20.66	2.11	0.59	0.06	-62.94	6.43
60	333.15	1.07	0.23	0.05	0.20	-0.04	0.13	-20.87	4.29	0.96	0.18	-62.64	12.88
65	338.15	0.49	0.22	-0.78	0.42	0.52	0.28	-36.97	9.66	2.29	0.83	-109.32	28.56
70	343.15	0.23	0.10	-1.53	0.39	1.04	0.27	-34.78	2.64	4.85	1.66	-101.37	7.69
75	348.15	0.12	0.03	-2.12	0.22	1.46	0.15	-27.82	8.50	8.47	1.78	-79.91	24.42
80	353.15	0.09	0.05	-2.55	0.51	1.78	0.35	-20.68	13.95	13.76	5.77	-58.57	39.51
85	358.15	0.03	0.02	-3.48	0.52	2.47	0.37	-46.99	17.34	35.62	18.84	-131.21	48.42
90	363.15	0.03	0.03	-3.86	0.98	2.77	0.70	-19.32	40.62	61.53	45.06	-53.20	111.86
